# The identification and functional analysis of CD8+PD-1+CD161+ T cells in hepatocellular carcinoma

**DOI:** 10.1038/s41698-020-00133-4

**Published:** 2020-10-30

**Authors:** Zhixuan Li, Bo Zheng, Xinyao Qiu, Rui Wu, Tong Wu, Shuai Yang, Yanjing Zhu, Xuan Wu, Shan Wang, Ziqi Gu, Siyun Shen, Mengchao Wu, Hongyang Wang, Lei Chen

**Affiliations:** 1 International Cooperation Laboratory on Signal Transduction, Eastern Hepatobiliary Surgery Institute, Second Military Medical University, 200438 Shanghai, P. R. China; 2 Fudan University Shanghai Cancer Center; Department of Oncology, Shanghai Medical College, Fudan University, 200032 Shanghai, China; 3 Department of Biliary Surgery I, Eastern Hepatobiliary Surgery Hospital, Second Military Medical University, Changhai Road 225, 200438 Shanghai, China; 4 Department of Laboratory Medicine, The Tenth People’s Hospital of Shanghai, Tongji University, 200072 Shanghai, China

**Keywords:** Tumour immunology, Diagnostic markers

## Abstract

Immunotherapy is a powerful therapeutic strategy for end-stage hepatocellular carcinoma (HCC). It is well known that T cells, including CD8+PD-1+ T cells, play important roles involving tumor development. However, their underlying phenotypic and functional differences of T cell subsets remain unclear. We constructed single-cell immune contexture involving approximate 20,000,000 immune cells from 15 pairs of HCC tumor and non-tumor adjacent tissues and 10 blood samples (including five of HCCs and five of healthy controls) by mass cytometry. scRNA-seq and functional analysis were applied to explore the function of cells. Multi-color fluorescence staining and tissue micro-arrays were used to identify the pathological distribution of CD8+PD-1+CD161 +/− T cells and their potential clinical implication. The differential distribution of CD8+ T cells subgroups was identified in tumor and non-tumor adjacent tissues. The proportion of CD8+PD1+CD161+ T cells was significantly decreased in tumor tissues, whereas the ratio of CD8+PD1+CD161− T cells was much lower in non-tumor adjacent tissues. Diffusion analysis revealed the distinct evolutionary trajectory of CD8+PD1+CD161+ and CD8+PD1+CD161− T cells. scRNA-seq and functional study further revealed the stronger immune activity of CD8+PD1+CD161+ T cells independent of MHC class II molecules expression. Interestingly, a similar change in the ratio of CD8+CD161+/ CD8+CD161− T cells was also found in peripheral blood samples collected from HCC cases, indicating their potential usage clinically. We here identified different distribution, function, and trajectory of CD8+PD-1+CD161+ and CD8+PD-1+CD161− T cells in tumor lesions, which provided new insights for the heterogeneity of immune environment in HCCs and also shed light on the potential target for immunotherapy.

## Introduction

Hepatocellular carcinoma (HCC) is one of the most common primary liver cancers, ranking the fourth leading cause of cancer-related death worldwide^1^. For patients in advanced stages, surgical resection does not completely cure the disease. Combination therapy with radiotherapy, chemotherapy, hormonal therapy, and targeted therapy is usually required^2–4^. However, benefits of treatment are not satisfactory. With rapid development of immunotherapy research, it is hoped that immunotherapy may grant more significantly therapeutic effects to patients with advanced HCC. Although some patients could benefit from anti-PD-1 and anti-CTLA-4 treatment, there is no sign that these therapies clearly ameliorate the prognosis^5^. Therefore, it is urgent need to understand the immune environment and functional status of immune cells in HCC more deeply.

Previous studies have been performed to map the immune microenvironment of liver cancer using single-cell sequencing^6^. Although the accumulation of CD8+PD-1+ T cells in HCC was observed with worse outcome clinically^7^, there is still a lack of deep phenotypic interrogation of heterogeneity subpopulation within CD8+PD-1+ T cells. PD-1, an inhibitory receptor on activated T cells and an important immune checkpoint therapeutic target, is used to reactivate cytotoxic T cells in cancer immunotherapy^8^. But the expression of PD-1 alone does not determine exhausted stage of T cells^9^, of which distinct subsets have different distribution patterns, phenotypes, and functions as well. Therefore, to explore the composition of CD8+PD-1+ T cells and identify critical exhausted subgroup is necessary for tumor immunotherapy.

Single-cell sequencing (scRNA-seq)^10^ and mass spectrometry (CyTOF)^11^, two complementary single-cell high-dimensional analysis methods, have increasingly been used to explore the transcriptional and functional changes in different types of cancers^12–14^. High-throughput sequencing at the single-cell level can help discover new potential functional subpopulations and expand understanding of tumor immune microenvironment. Existing studies also confirmed that high-dimensional mass spectrometry methods can not only help identify the optimal combination of currently available immuno-oncology drugs but also identify potential new prognostic markers, which make cancer immunotherapy more accurate, safe, and effective^15^.

In our study, we applied mass cytometry to process immune cells in matched tumor, non-tumor adjacent tissues, and blood samples. The regional distribution and functional differences of CD8+PD-1+CD161+/− T cells were firstly identified. Enrichment of CD8+PD-1+CD161+ T in tumor nodule indicates a better prognosis clinically. Mechanically, co-expression of CD161 and interleukin-7R (IL-7R) maintains the proliferation capacity of CD8+PD-1+ T cells instead of “exhausted” state, at least partly, through enhancing the expression of IL-2, tumor necrosis factor-α (TNF-α), and periforin (PRF).

## Results

### Distribution landscape of T cells between HCC and non-HCC tissues

**Fig. 1:**
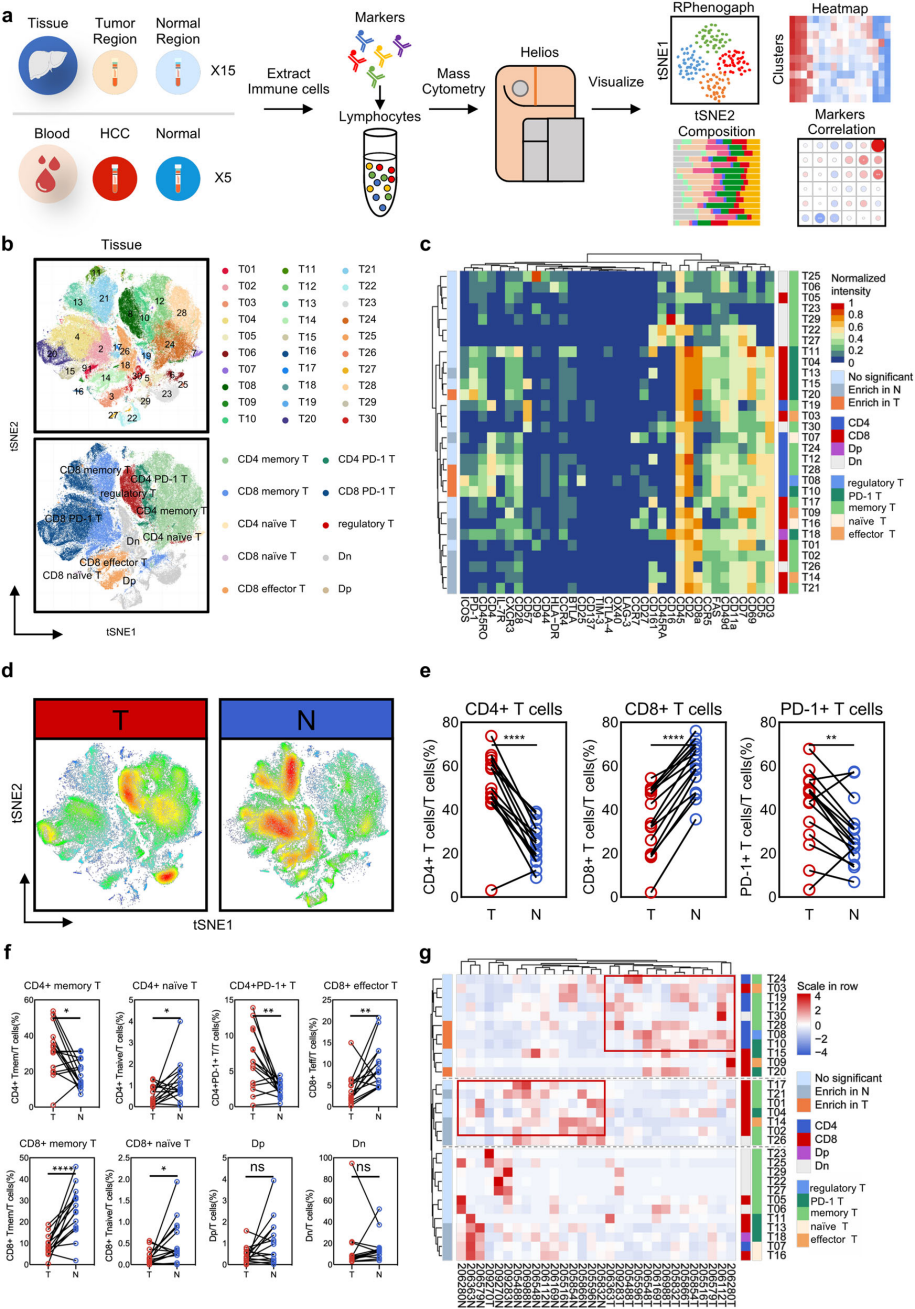
Distribution of T cells showed spatial difference in HCC. **a** Single-cell mass cytometry workflow. **b** Thirty clusters and 10 annotation subpopulations T cells tSNE plot. **c** Thirty-five markers heat map with enrichment differences between T (tumor tissues) and N (normal tissues). **d** Heat distribution diagram of all T cells. **e** Distribution difference of CD4+, CD8+, and PD-1+ T cells. **f** Significant differential enrichment in T and N regions (*p < 0.05, **p < 0.01, ***p < 0.001, ****p < 0.0001). **g** Clustering 15 paired T and N samples.

We set up a patient cohort consisting 15 HCC patients (Supplementary Table 1) and extracted T cells from paired tumor region (T) and adjacent normal region (N) tissues. Mass cytometry was applied to acquire multi-parametric information on protein level from these samples (Supplementary Table 2). Entire workflow is shown in Fig. 1a. We collected approximately 20,000,000 cell counts (average 450,000 cell counts per sample), of which 150,000 T cells (average 5000 cell counts per sample) were extracted for tSNE visualization and further analysis (Supplementary Fig. 1a).

We visualized high-dimensional data through dimensionality reduction to obtain a tSNE map with 30 clusters (T01–T30) and 10 annotation subpopulations (Fig. 1b and Supplementary Fig. 1b). Clusters and phenotypes of T cells are shown in heat map (Fig. 1c and Supplementary Fig. 1c), including CD25+IL-7R− regulatory T cells (T08), PD-1+ T (T04, T10, T11, T13, T15, T18 and T20), CD45RO+memory T cells (T01, T02, T05, T06, T12, T17, T19 and T21–T30), CD45RA+CCR7+naïve T cells (T07 and T16), and CD45RA+CCR7− effector T cells (T03, T09, and T14). We tested and labeled differences between T and N regions (Fig. 1c and Supplementary Fig. 1d). T08, T10, T20, and T28 groups in T regions are significantly higher than N regions, while trends of T02, T07, T13, T14, T16, T18, T21, and T26 are exactly reverse. Despite difference across individuals, proportion of CD4+, CD8+, and PD-1+ T cells show significantly different distribution between tumor or non-tumor tissues (Fig. 1d, e and Supplementary Fig. 1e). Interestingly, we find that not all PD-1+ T cells are enriched in the tumor area. T13 with CD161 expression even show the opposite trend (Fig. 1c and Supplementary Fig. 1d). At the same time, CD161+ effector T cells and some CD161+ memory T cells also are enriched in N. Overall, composition of T cells in T and N regions is comparable and most of infiltrating T cells in tissue are memory T cells (Supplementary Fig. 1f). CD4+ memory T cells, CD4+PD-1+ T cells, and regulatory T cells were enriched in T regions, and there was no statistical difference in the distribution of CD8+PD-1+ T cells, Dp, and Dn. The remaining subgroups accounted for relatively higher in N regions (Fig. 1f and Supplementary Fig. 2b). As the clustering relationship shown in Fig. 1g, compared with N regions, the immune microenvironment in T regions of different samples has changed dramatically.

### The enrichment of CD8+PD-1+CD161− T cells in HCCs

**Fig. 2:**
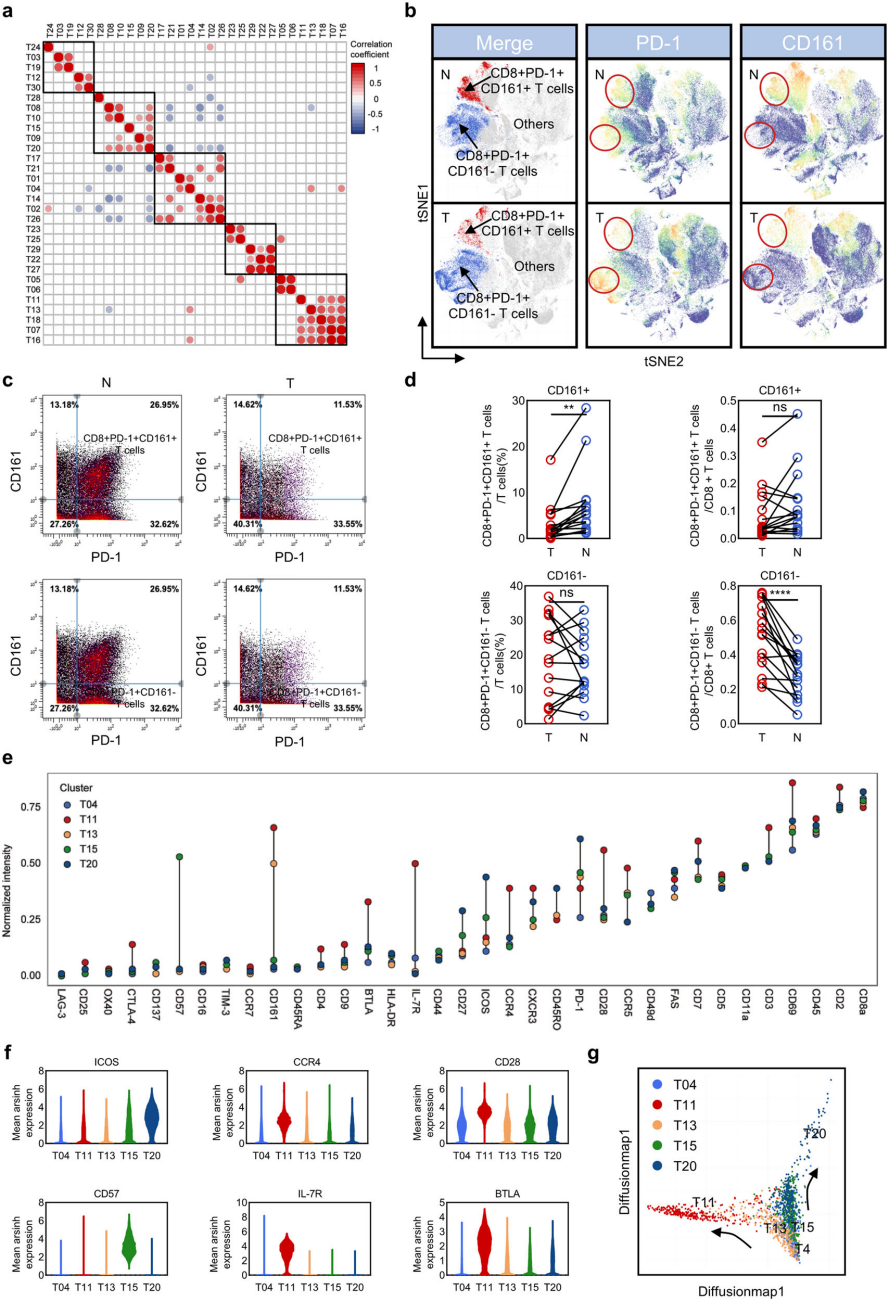
Changes in the proportion of CD8+PD-1+CD161+/− T cells and phenotype difference. **a** T cells correlation matrix. **b** Characteristic markers tSNE plot (PD-1 and CD161). **c**, **d** Reverse changes of CD8+PD-1+CD161+/CD8+PD-1+CD161− T cells in T and N, figure **c** is pre-gated with CD8. **e** Thirty-five markers mean expression of CD8+PD-1+CD161+ T cells (T11 and T13) and CD8+PD-1+CD161− T cells (T04, T15, and T20). **f** Differential expression of ICOS, CCR4, CD28, CD57, IL-7R, and BTLA. **g** Diffusion map of CD8+PD-1+ T cells.

As shown in Fig. 2a, significant positive correlation among CD8+PD-1+CD161+ T cells (T11 and T13), Dp cells (CD4 and CD8 double-positive T cells, T18), and naïve T cells (T07 and T16) was observed, while CD8+PD-1+CD161− T cells (T20) were found positively correlated with regulatory T cells (T08). In comparison with CD8+PD-1+CD161+ T cells, CD8+PD-1+CD161− T cells were found dominantly located in tumor tissues (Fig. 2b–d). In addition, the proportion of IL-7R-CD25+ regulatory T cells was higher in tumor than in adjacent non-tumor tissues (Supplementary Fig. 2a, b).

To fully identify phenotypic characteristics of CD8+PD-1+CD161+ T cells (T11 and T13) and CD8+PD-1+CD161− T cells (T04, T15, and T20), we further compared differential expression level of 35 surface markers (Fig. 2e, f). As the dramatically increased expression of CD57, an irreversible marker of terminal differentiation indicates that T15 is an “older” subset^16^. A higher expression level of ICOS indicates that T20 is activated by PI3K signal and then transferred to exhausted stage through PD-1/SHP2 or PTEN signal^17,18^. It is worth emphasizing that T20 possesses the highest level of PD-1. Overall, the PD-1 expression level of CD8+CD161− T cells (T15 and T20, except T04) is also higher than that of CD8+CD161+ T cells (T11 and T13) (Fig. 2c). The highest expression of IL-7R, CCR4, and CD28 in T11 indicates potential proliferative activity, together with the higher level of BTLA and CCR4 implying the possible feedback regulation and Th2 characteristics. In addition, diffusion-map analysis further implied two fates of CD8+PD-1+ T cells: both of them are generated from T04 cluster, one is T04-T13-T11 trajectory (CD8+PD-1+CD161+, with proliferative and active phenotype) and the other is T04-T15-T20 trajectory (CD8+PD-1+CD161−, with gradually exhausted and older phenotype) (Fig. 2g).

### CD8+PD-1+CD161+ T cell subsets have stronger cytotoxicity and proliferative capacity

**Fig. 3:**
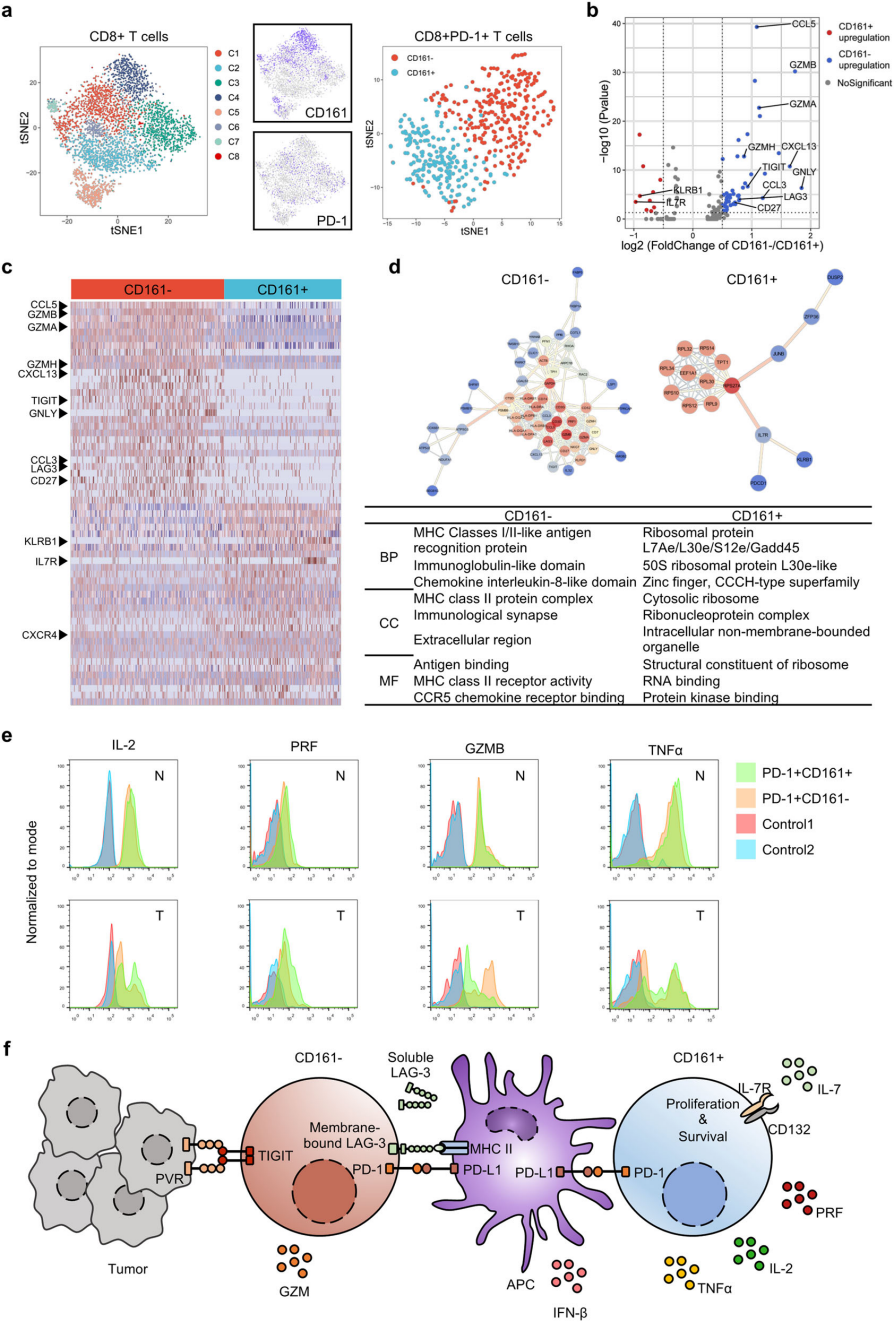
CD161+ T cell subsets have stronger cytotoxicity and proliferative capacity. **a** CD161+ and CD161− two subsets of CD8+PD-1+ T cells. **b**, **c** Volcanic and heat map showing the differential expression. **d** GO enrichment analysis and proteins interaction (**e**) secretion level of IFN-γ, TNF-α, IL-2,GNLY, PRF, and GZMB in indicated subsets of T cells. Mononuclear cells were isolated from human HCC tumor region (T) and the surrounding non-tumor region (N) areas and stimulated with PMA+ Ionomycin+ for 4.5 h. Then, the cells were subjected to staining with indicated markers. **f** Potential regulation mechanism.

We then performed scRNA-seq (Fig. 3a) to explore the diversity of CD8+ T cells and classified eight clusters with the heterogenic expression of PD-1 and CD161. A total of 193 genes were identified with significantly expressional level between CD8+PD-1+CD161+ and CD8+PD-1+CD161− T cells, 42 of which were highly expressed in CD8+PD-1+CD161+ T cells and 151 were increased in CD8+PD-1+CD161− T cells. It is worth noting that *CCL5*, *GZMB*, *GZMA*, *GZMH*, *CXCL13*, *TIGIT*, *GNLY*, *CCL3*, *LAG3*, and *CD27* were up-regulated in CD8+PD-1+CD161− T cell subset, while *IL-7R* showed significant up-regulation in CD8+PD-1+CD161+ T cell subset. Although CD8+PD-1+CD161− T cells showed a higher level of secretory cytotoxin mRNAs, synergetic expression of PD-1, TIGIT, and LAG3 indicated their exhausted status (Fig. 3b, c). GO annotation enrichment and protein interaction analysis revealed that CD8+PD-1+CD161− T cell subset possessed on MHC class II molecules whereas synthesis and proliferation-related terms were enriched in CD8+PD-1+CD161+ T cell subset (Fig. 3d).

We then isolated primary CD8+PD-1+CD161+/− T cells from T and N regions, and compared secretion levels of IFN-γ, TNF-α, IL-2, GNLY, PRF, and GZMB in CD8+PD-1+CD161+ T cells and CD8+PD-1+CD161− T cells after stimulating with PMA+Inomycin+. As shown in Fig. 3e and Supplementary Fig. 3a, the levels of TNF-α, IL-2, and PRF were increased in CD8+CD161+ subset. Interestingly, in N region, the presence of PD-1 in CD8+CD161+ T cells resulted in higher levels of TNF-α, IL-2, and PRF in comparison with CD8+CD161+PD-1− T cells, while CD8+CD161+PD-1− T cells showed opposite trend of TNF-α and IL-2 levels in tumor tissues (Fig. 3e), indicating that CD8+PD-1+CD161+ T cells adjacent to tumor tissue have stronger immunity activity (Fig. 3f). These data suggested that only PD-1 expression cannot determine CD8+ T cells functional status and exhausted stage is the result of T cell educated by tumor microenvironment. Co-expression of CD161 and IL-7R is necessary for the cytotoxic capacity of CD8+PD-1+ T cells.

### The higher infiltration level of CD8+PD-1+CD161+ T cells indicates better prognosis

**Fig. 4:**
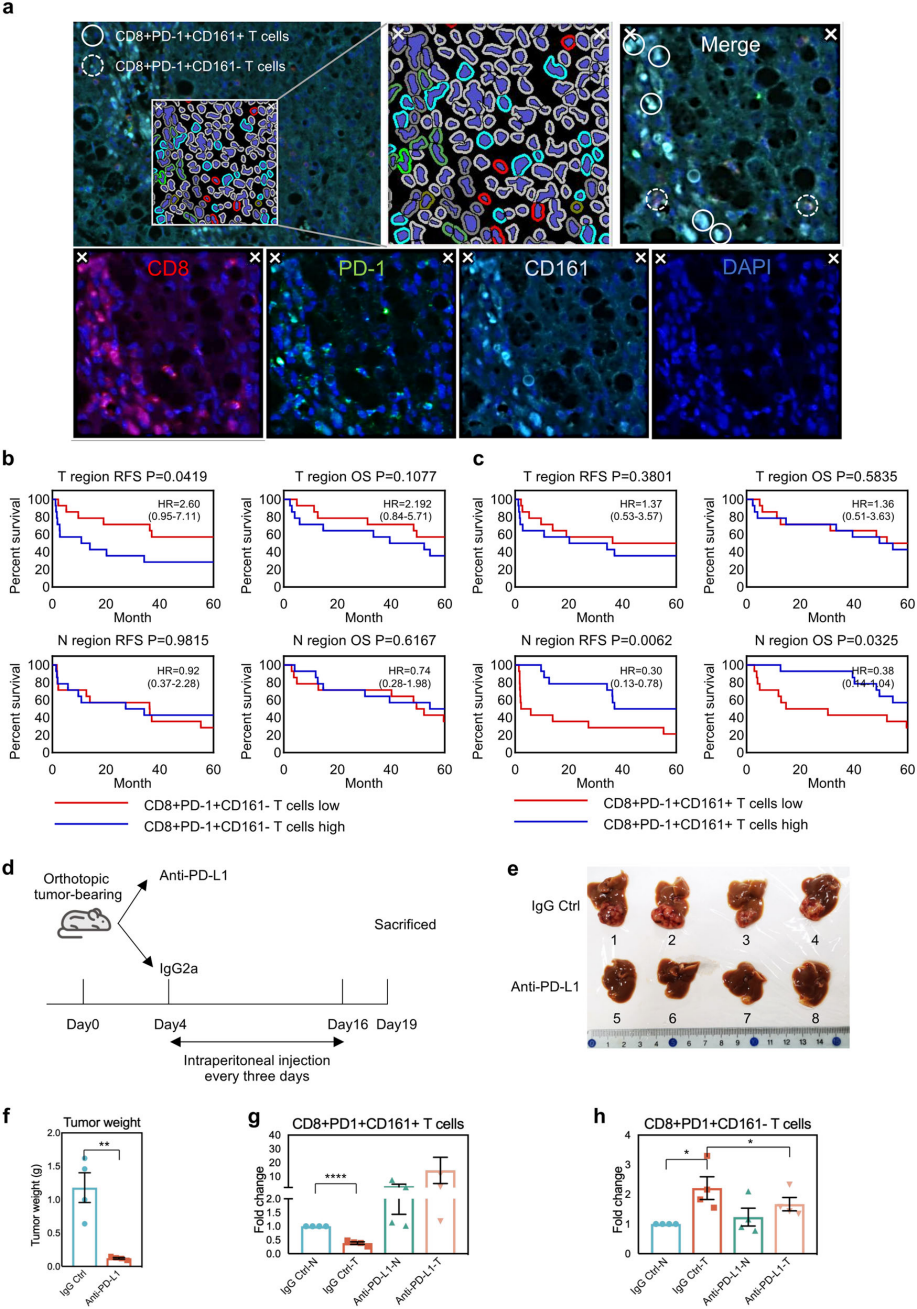
The higher infiltration level of CD8+PD-1+CD161+ T cells indicates better prognosis. **a** Opal multiplex immunohistochemical staining. **b** RFS (recurrence-free survival) and OS (overall survival) of CD8+PD-1+CD161− T cells in the infiltration high group (*n* = 14) and low group (*n* = 14), HR (hazard ratio) with 95% CI. **c** RFS and OS of CD8+PD-1+CD161+ T cells in infiltration high group (*n* = 14) and low group (*n* = 14). **d** Work flow of mouse anti-PD-L1 treatment. **e** General view of tumors. **f** Tumor weight. **g**, **h** Distribution of CD8+PD-1+CD161+ T cells and CD8+PD-1+CD161− T cells in T and N region before and after anti-PD-L1 treatment. (The data of each group was normalized according to the value of IgG Ctrl-N.) N non-tumor region, T tumor region.

We then applied multiplex immunofluorescence staining (Fig. 4a) to evaluate whether the enrichment of CD8+PD-1+CD161+ or CD8+PD-1+CD161− T cells has potential correlation with patient prognosis. As shown in Fig. 4b, patients with CD8+PD-1+CD161− T cells enriched in tumor tissues had shorter recurrence-free survival (RFS, high vs. low = 28.7 months vs. 54.6 months, *P* = 0.0419) than patients with less CD8+PD-1+CD161− T cells, whereas no difference was observed for overall survival (OS) (Fig. 4b). Meanwhile, higher level of CD8+PD-1+CD161+ T cells in non-tumor adjacent tissues indicates the favorable prognosis after resection (RFS, high vs. low = 54.9 months vs. 24.3 months, *P* = 0.0062; OS, high vs. low = 66.1 months vs. 37.5 months, *P* = 0.0325) (Fig. 4c). Neither CD8+PD-1+CD161+ T cells in tumor nor CD8+PD-1+CD161− T cells in non-tumor adjacent tissues showed potential correlation with prognosis (Fig. 4b, c). In addition, we compared the change of these two types of cells under anti-PD-L1 treatment condition in an orthotopic tumor-bearing model in mice and the immune cells were subject to flow sorting (Fig. 4d and Supplementary Fig. 4c). In the control group, the distribution of these two types of T cells is consistent with our previous results obtained in human samples (Fig. 4g, h). Anti-PD-L1 treatment significantly reduced tumor burden (Fig. 4e, f) and reversed the downward trend of CD8+PD-1+CD161+ T cells in the T region, while for CD8+PD-1+CD161− T cells, it reduced the enrichment degree in T region (Fig. 4g, h). In view of the fact that patients with CD8+PD-1+CD161+ T cells enriched next to the cancer have better outcomes and the anti-PD-L1 treatment of mice, and it can be considered as a potential prognostic indicator and may benefit from anti-PD-L1 treatment.

### Abundance change of CD8+CD161 +/− T cells in blood samples

**Fig. 5:**
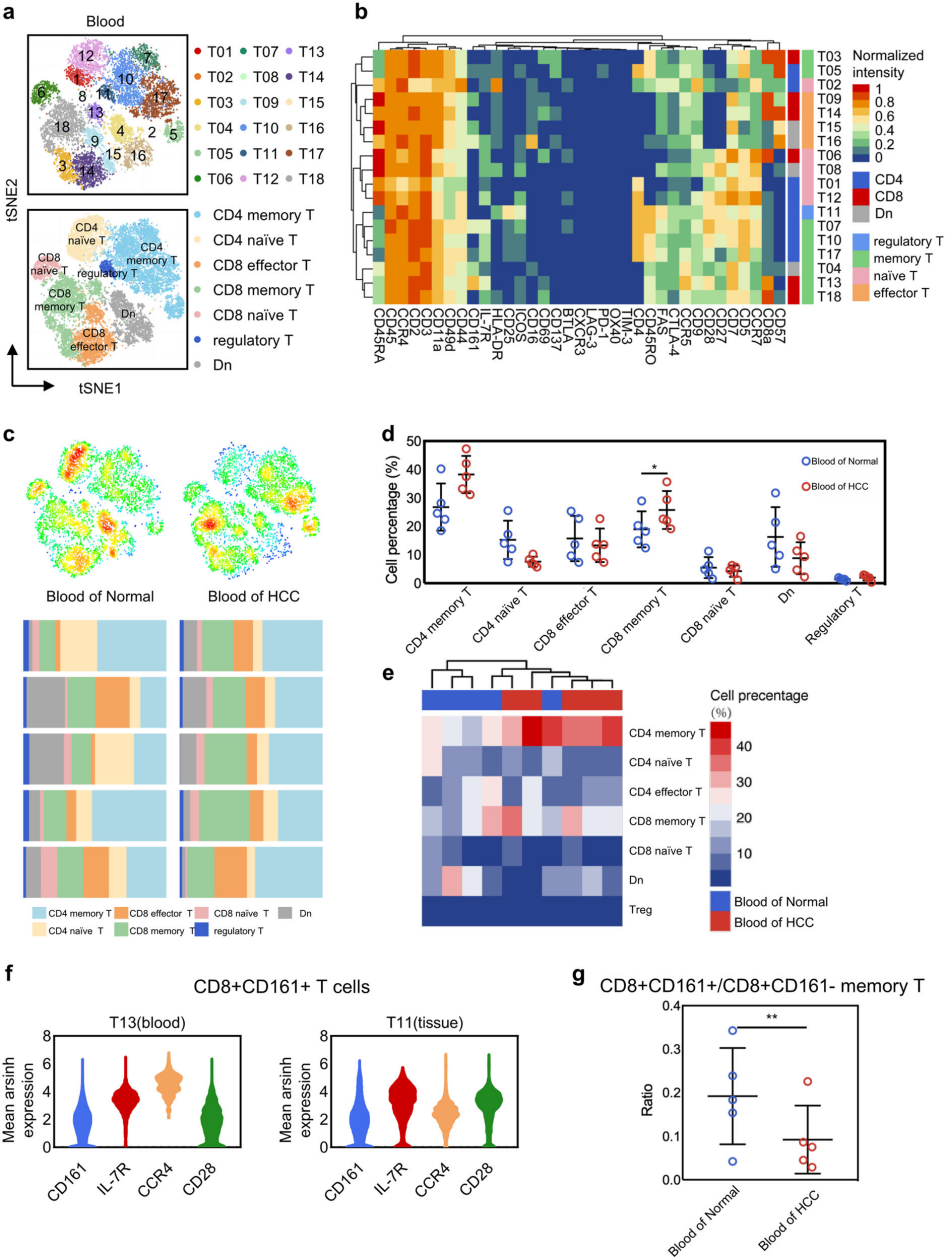
Abundance change of CD8+CD161+/ CD161− T cells in different human blood. **a** tSNE plot of 18 T cell clusters and 7 annotation subpopulations. **b** Heat map of 35 markers. **c** Distribution and composition of seven annotation T cell clusters in Normal and HCC. **d** Enrichment difference scatter plot. **e** Clustering abundance heat map. **f** Similar expression pattern of CD8+CD161+ T cells in blood and tissues. **g** Differential abundance of CD8+CD161+/ CD8+CD161− memory T cells in normal and cancer patient blood samples.

In some autoimmune diseases, CD8+CD161+ T cells are enriched in diseased tissues and blood samples simultaneously^19,20^. We here attempted to explore whether the occurrence of tumors will lead to the change of immune cell composition in blood. A total of 12,000 cells collected from blood samples were analyzed (average 1200 cells per sample), and identified 18 clusters, including regulatory T cells, memory T cells, naïve T cells, and effector T cells according to their classical molecular features (Fig. 5a, b and Supplementary Fig. 5a). As shown in Fig. 5c, d, a higher level of CD8+ memory T cells was found in blood samples of HCC. Besides CD8+ memory T cells, obvious differences of cluster distribution in blood were observed among healthy and HCC cases (Fig. 5e). Interestingly, similar expression pattern of CD8+CD161+ T cells was found in both tissue and blood samples (Fig. 5f). In accordance with tumor tissues, the ratio of CD8+CD161+ memory T cells vs CD8+CD161+ memory T cells showed dramatic reduction in blood samples collected in HCC cases (Fig. 5g). Taken together, these results suggested that it is possible to non-invasively monitor tumor development and progression through evaluating significant pattern of immune cells in blood instead of tumor tissues.

## Discussion

Enrichment of CD8+CD161+ T cells and its pro-inflammatic activity have been documented in several types of diseases^19–21^. CD8+CD161+ T cells were phenotypically defined as CCR6+ and IL-17+, and the production of IFN-γ could promote autoimmune and inflammatory responses. It was also found that these cells could secrete IL-22, indicating the potential role for anti-bacteria and other viruses^22^. Interestingly, CD8+CD161+ T cells also have specific affinity for liver tissue because of upregulated CXCR6(*23*). This may depend on the complex environment of the liver and some infections of the Hepadnaviridae (e.g. HBV and HCV)^23^. However, the underlying role of CD8+PD-1+CD161+ T cells in HCC remains unclear.

Our study here attempted to explore the different functions of CD8+PD-1+CD161+ and CD8+PD-1+CD161− T cells. Unlike classic distribution of CD8+PD-1+ T cells, CD8+PD-1+CD161+ T cells are preferentially dwelled in N region, whereas the enrichment of CD8+PD-1+CD161+ T cells in tumor in some HCC cases shows positive correlation with better prognosis. Mechanically, increased expression of both IL-7R in CD8+PD-1+CD161+ T cells renders the higher proliferation activity rather than CD8+PD-1+CD161− T cells. Meanwhile, the stronger capability to secrete PRF and TNF-α rather than GZMB through MHC class II molecules might be responsible for the anti-tumor activity of CD8+PD-1+CD161+ T cells.

It is useful to identify non-invasive biomarkers for both cancer diagnosis and prognosis prediction. Previous studies have documented that the distinct distribution of immune cells in blood can be used to monitor the drug response before or after treatment^24^. In our study, we observed the reduced ratio of CD8+CD161+ vs CD8+CD161− memory T cells in blood samples in HCC patients as compared with healthy control. Further study with a larger cohort should be warranted to evaluate its potential usage for not only tumor diagnosis but also relapse surveillance.

## Methods

### Patients and sample

Paired tumor and adjacent non-tumor tissues are collected from 15 HCC patients (the clinical characteristics of each patient are listed in Supplementary Table 1) undergoing liver resection in the Eastern Hepatobiliary Surgery Hospital. A total of 10 blood samples from healthy people and HCC patients are obtained through routine physical examination and voluntary donation. One case (205488) of mixing TILs or NILs was subjected to scRNA-seq to further require difference in transcriptome levels. Leukocytes were extracted from the central region of the tumor, adjacent non-tumor regions (taken > 0.6 cm distal to the macroscopic malignant-benign boundary), and peripheral blood are anonymously coded according to the regional ethics guidelines. This study of human specimen collection was approved by the Ethics Committee of Eastern Hepatobiliary Surgery Hospital. All human tissue and blood samples were obtained from subjects who preoperatively provided written informed consent permitting the investigational use of their tissue.

### Mice

Male C57BL/6JCrl mice aged 6–8 weeks were purchased from Model Animal Resource Information Platform (Nanjing, China). Mice were housed under specific pathogen-free conditions at Eastern Hepatobiliary Surgery Hospital. The orthotopic HCC mouse model was built by injecting Hepa1–6 cells (3 × 10^6^/injection) to the left liver lobe of C57BL/6 mouse at 8 weeks. Five days after implantation, mice were randomly separated into the control group and the treatment group. The control group was injected with Rat IgG2a isotype control (200 μg/injection, i.p., clone 1-1, Leinco Technologies, Inc.), and the treatment group was injected with anti-PD-1 (200 μg/injection, i.p., clone RMP1–14, Leinco Technologies, Inc.)) once every 3 days for 2 weeks. All experimental protocols were approved by the Ethics Committee of Eastern Hepatobiliary Surgery Hospital.

### Extraction of leukocytes

*Digestive enzymes*: collagenase IV, deoxyribonuclease type I, and hyaluronidase type V mixture dissolved in RPMI containing 10% serum.

*Tissue digestion*: The tissue washed with HBSS (Hank’s balanced salt solution) was chopped and digested with the above digestive enzymes, shaken at 37 °C for 60 min, and then filtered through a 300-mesh filter. Finally, the filtered mixture was collected in 50 ml centrifuge tubes.

*Density gradient centrifugation*: The collected mixture was centrifuged at 450*g* for 8 min, then reprecipitated with HBSS, and centrifuged for 1 min with 50*g*. The supernatant was added to the lymphocyte fluid, centrifuged at 450*g* for 25 min, and the concentrated leukocytes were aspirated from the intermediate layer. When processing blood samples, tissue digestion is skipped and density gradient centrifugation is performed directly.

### Mass cytometry

Fifteen groups of TILs and NILs and ten samples of leukocytes from peripheral blood were obtained as described above and subjected to CyTOF analysis. A total of 35 immune antibodies (Supplementary Table 2) were used to distinguish immune cell subsets. Preconjugated antibodies are purchased directly from Biolegend, USA. Leukocytes were washed and stained with 10 mM cisplatin for 2 min to identify live/dead cells, and incubated with metal-conjugated surface-membrane antibodies for 30 min at 37 °C. The cells were then fixed and added to a cell intercalation (mixture of fix perm buffer and iridium) for overnight. Finally, visual analysis was performed with a Helios mass cytometer (Fludigm, USA).

### Data processing

The signals were normalized using the EQ Four Element Calibration Beads according to the supplier’s guidebook. Each sample was collected from 250,000 to 500,000 cell events, and the exported FCS file was uploaded to Cytobank for manual gating of cell subpopulations. To further analyze the data in depth, we applied Cytofkit program on R to randomly sample the FCS file, perform clustering with Renograph algorithm, and reduced the dimensionality to construct the tSNE image for visual depiction and other analysis.

### Flow cytometry analysis

Mononuclear cells were isolated from human HCC tumor region (T) and the surrounding non-tumor region (N) with Lymphoprep (STEMCELL, Germany) following the manufacturer’s instructions. Cells were collected and washed with HBSS. After stimulating with Phorbol 12-myristate 13-acetate (25 ng/ml, PeproTech), Ionomycin (1 μg/ml, PeproTech), and Brefeldin A (10 μg/ml, PeproTech) for 4.5 h, the samples were stained with surface markers mouse anti-human Alexa Fluor 700-conjugated CD3 (clone UCHT1, BD Pharmigen), BB515-conjugated CD8 (clon3 RPA-T8, BD Horizon), and Percp/Cyanine 5.5-conjugated PD-1 (clone EH12.2H7, Biolegend). Cells were then incubated with transcription factor fixation/permeabilization working solution (Biogems) for 30 min at 4 °C, washed with permeabilization working solution (Biogems), and stained with either mouse anti-human BV421 conjugated IL-2 (clone 5344.111, BD Horizon), BV650 conjugated TNF-α (clone MAb11, BD Horizon), PE-conjugated IFN-γ (clone 4S.B3, BD Pharmigen), PE-CF594-conjugated Perforin (δG9, BD Horizon), PE-conjugated Granzyme B (GB11, BD Pharmigen), or PE-conjugated Granulysin (DH2, Biolegend). After washing with permeabilization working solution, the samples were subjected to flow cytometry analysis (BD LSRFortessa). Lymphocytes were isolated from orthotopic mouse HCC tumor region (T) and the surrounding non-tumor region (N) in the left lobe with Lymphoprep (STEMCELL, Germany) following the manufacturer’s instructions. After staining with surface markers Alexa Fluor 700-conjugated CD45 (clone 30-F11, BD Bioscience), BB700-conjugated CD3e (clone 145-2c11, BD Bioscience), FITC-conjugated CD8a (clone 53-6.7, BD Bioscience), PE-conjugated PD-1 (clone J43, BD Bioscience), and BV421-conjugated CD161a (clone 10/78, BD Bioscience), the samples were subjected to flow cytometry analysis (BD LSRFortessa). Lymphocytes were isolated from orthotopic mouse HCC tumor region (T) and the surrounding non-tumor region (N) in left lobe with Lymphoprep (STEMCELL, Germany) following the manufacturer’s instructions. After staining with surface markers Alexa Fluor 700-conjugated CD45 (clone 30-F11, BD Bioscience), BB700-conjugated CD3e (clone 145-2c11, BD Bioscience), FITC-conjugated CD8a (clone 53-6.7, BD Bioscience), PE-conjugated PD-1 (clone J43, BD Bioscience), and BV421-conjugated CD161a (clone 10/78, BD Bioscience), the samples were subjected to flow cytometry analysis (BD LSRFortessa). The data were further analyzed with FlowJo vX.07 software (Tree Star).

### Multiplex immunofluorescence tissue staining

Three tissue micro-arrays containing T/N samples from 28 patients were stained with Opal Multiplex Immunohistochemistry Assay Kit (Perkin-Elmer) and images were acquired using a Vectra 3.0 Pathology Imaging System microscope (Perkin-Elmer). Slides were deparaffinized and rehydrated, and antigen retrieved using microwave treatment (for 45 s at 100% power and 15 min at 20% power) antibodies used were anti-CD8, anti-PD-1, and anti–CD161. Detection dye for each antibody was Opal570 dye (CD8), Opal690 dye (PD-1), and Opal520 dye (CD161). DAPI was used as a nuclear counterstain. The digital images were analyzed with Halo™ Image Analysis software (indica labs). Antibodies used in this experiment and the corresponding dilution ratio are listed in Supplementary Table 1.

### scRNA-seq

We used CountessII Automated Cell Counter (Thermo Fisher Scientific, USA) to count cells waiting to be tested and adjusted concentration to an ideal concentration of 1 × 10^6^/ml. Then, cDNA was marked by 10x GemCode Technology. Gel beads containing barcode information were first mixed with cells and enzymes. Droplets were flowed into the reservoir and were collected and then dissolved and released primer sequences for reverse transcription. cDNA was used as templates to amplify PCR. A sequencing library was constructed by mixing products containing barcode amplification information in each droplet. First, DNA fragments were broken into 200–300 BP fragments by a Biorupter Ultrasound Fragmentation Instrument. Next, DNA library was amplified by PCR with sequencing connector P5 and sequencing primer R1. Finally, prepared samples were subjected to the 10× single-cell sequencing analysis platform.

### Reporting summary

Further information on research design is available in the Nature Research Reporting Summary linked to this article.

## Supplementary information

Supplementary Information

Reporting Summary

## Data Availability

Mass cytometry (CyTOF) and processed single-cell RNA sequencing data (.csv files), generated during the current study, are publicly available in the Mendeley Data repository: 10.17632/ttccnvj.5^25^. The raw single-cell RNA sequencing data (fastq files) are publicly available in Genome Sequence Archive (https://bigd.big.ac.cn/gsa/) under the accession number CRX075195^26^. Supplementary Figures 1–5 and Supplementary Tables 3–8 are available in the figshare repository: 10.6084/m9.figshare.12957425^27^. Patient survival data and tissue microarray data are not publicly available in order to protect patient privacy, but will be made available to researchers on reasonable request from the corresponding author H.W., email address: hywangk@vip.sina.com.

## Code availability

Our CyTOF and scRNA-seq analysis pipelines and codes can be obtained from the original author’s open source R package and script on Bioconductor. The default standard parameters are used in the analysis process.
