# Supplementary material for: The identification and functional analysis of CD8+PD-1+CD161+ T cells in hepatocellular carcinoma
Source: NPJ Precis Oncol. 2020 Oct 30;4:28. doi: 10.1038/s41698-020-00133-4 (PMC7599220; doi:10.1038/s41698-020-00133-4)
Supplement: Supplementary file 1 — Supplementary Information [file 41698_2020_133_MOESM1_ESM.pdf]

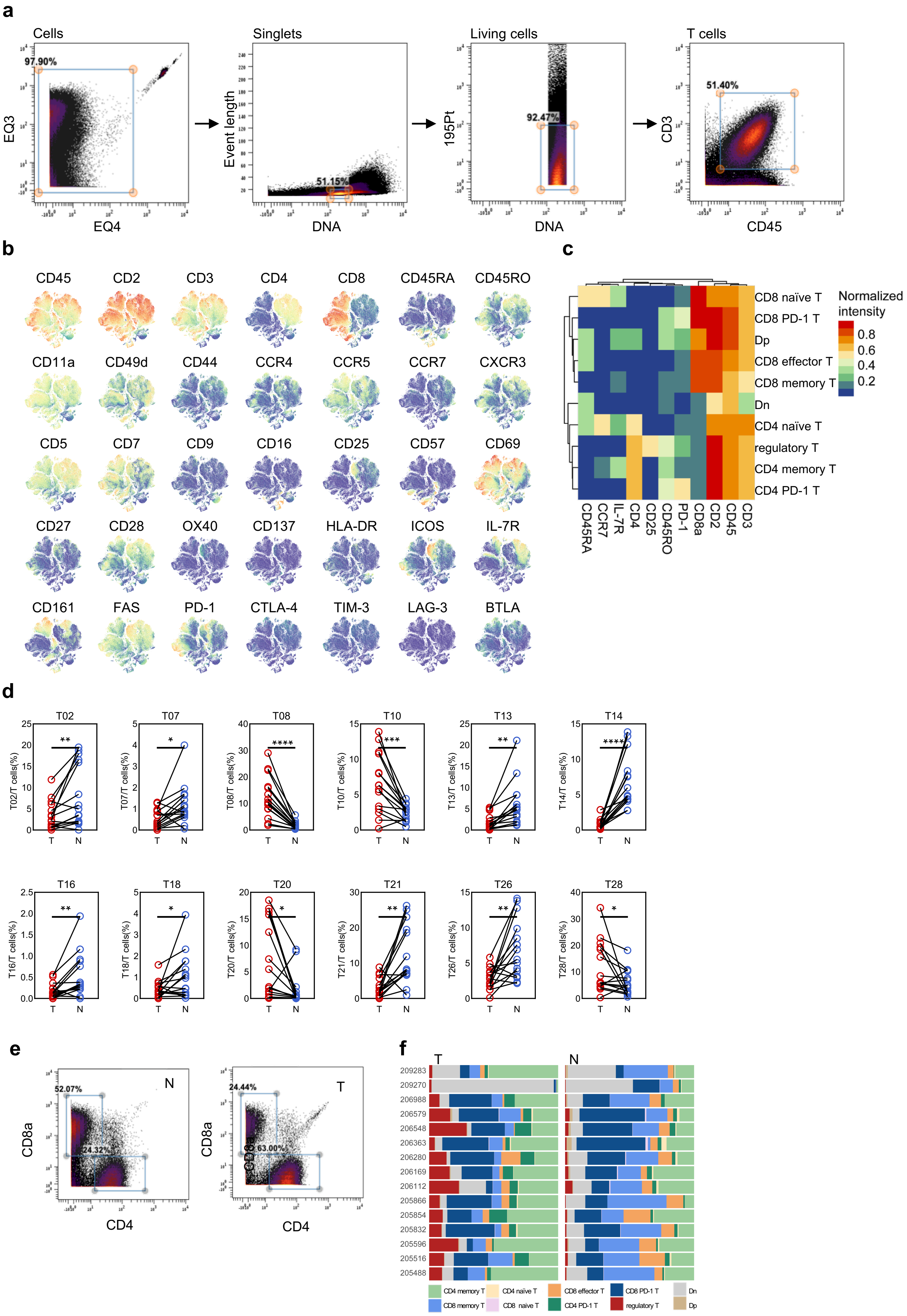

a
Cells
97.90%
EQ3
EQ4
Singlets
51.15%
Event length
DNA
Living cells
92.47%
195Pt
DNA
T cells
51.40%
CD3
CD45
b
CD45 CD2 CD3 CD4 CD8 CD45RA CD45RO
CD11a CD49d CD44 CCR4 CCR5 CCR7 CXCR3
CD5 CD7 CD9 CD16 CD25 CD57 CD69
CD27 CD28 OX40 CD137 HLA-DR ICOS IL-7R
CD161 FAS PD-1 CTLA-4 TIM-3 LAG-3 BTLA
c
CD8 naïve T
CD8 PD-1 T
Dp
CD8 effector T
CD8 memory T
Dn
CD4 naïve T
regulatory T
CD4 memory T
CD4 PD-1 T
CD45RA CCR7 IL-7R CD4 CD25 CD45RO PD-1 CD8a CD2 CD45 CD3
Normalized intensity
0.8
0.6
0.4
0.2
d
T02 T07 T08 T10 T13 T14
T02/T cells(%) T07/T cells(%) T08/T cells(%) T10/T cells(%) T13/T cells(%) T14/T cells(%)
** * **** *** ** ****
T16 T18 T20 T21 T26 T28
T16/T cells(%) T18/T cells(%) T20/T cells(%) T21/T cells(%) T26/T cells(%) T28/T cells(%)
** * * ** ** *
T N
e
N
52.07%
24.32%
T
24.44%
63.00%
CD8a
CD4
f
T
N
209283
209270
206988
206579
206548
206363
206280
206169
206112
205866
205854
205832
205596
205516
205488
CD4 memory T
CD4 naïve T
CD8 effector T
CD8 PD-1 T
Dn
CD8 memory T
CD8 naïve T
CD4 PD-1 T
regulatory T
Dp

Supplementary Fig. 1 (a) Gating T cell subsets on Cytobank. (b) tSNE plot of 35 markers. (c) Characteristic markers heat map of 10 annotation subpopulations (d) Significant differential enrichment in T and N regions (∗, $p<0.05$, ∗∗, $p<0.01$, ∗∗∗, $p<0.001$, ∗∗∗∗, $p<0.0001$). (e) Proportion differences of CD4+/CD8+ T cells in T and N regions. (f) Immune cells composition in T and N. (T: left, N: right).

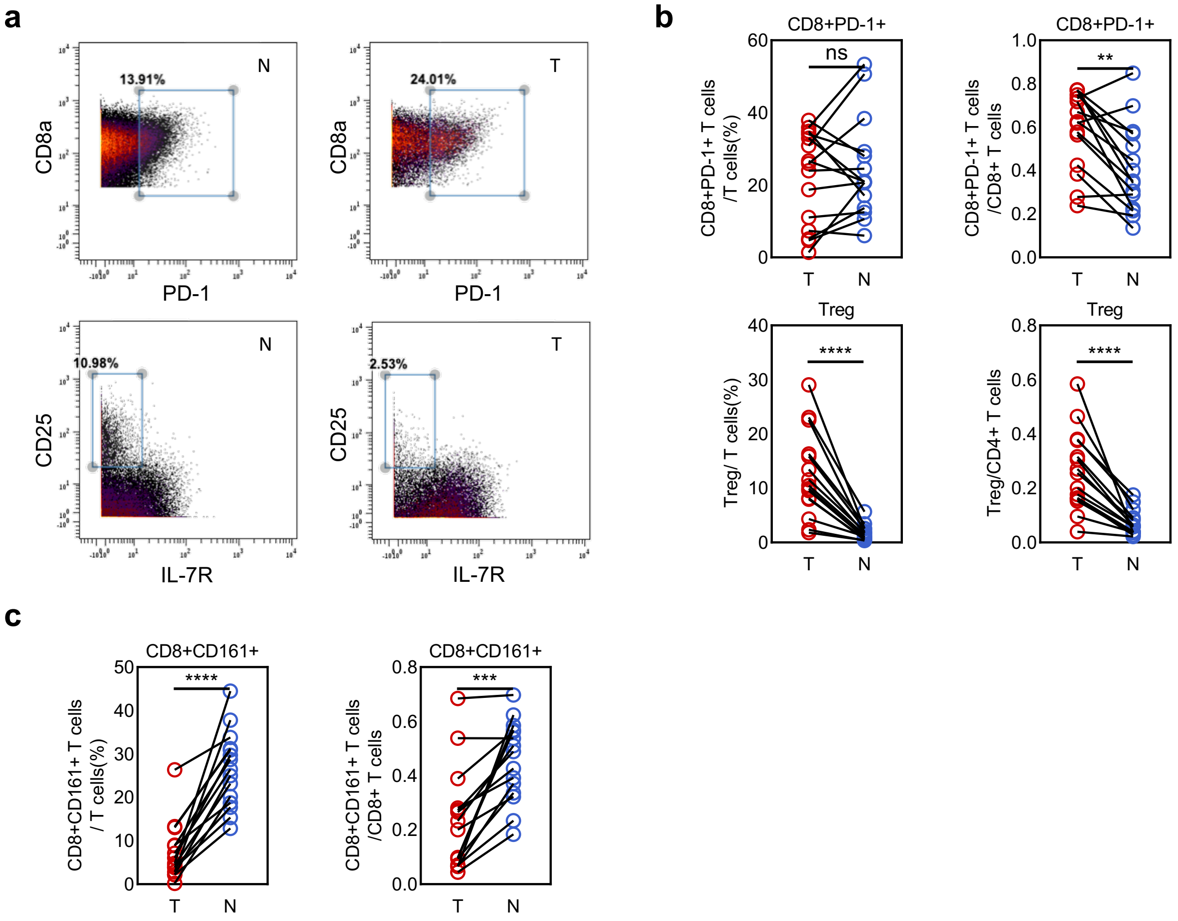
a
N
T
13.91%
24.01%
CD8a
PD-1
10.98%
2.53%
CD25
IL-7R
b
CD8+PD-1+
ns
CD8+PD-1+ T cells /T cells(%)
**
CD8+PD-1+ T cells /CD8+ T cells
Treg
****
Treg/ T cells(%)
Treg/CD4+ T cells
c
CD8+CD161+
****
CD8+CD161+ T cells / T cells(%)
***
CD8+CD161+ T cells /CD8+ T cells

Supplementary Fig. 2 (a, b) Distribution difference of CD8+PD-1+ T cells and Treg.

(c) Distribution differences of CD8+CD161+ T cells.

a

		PD-1+			PD-1-			CD161+			CD161-		
		CD161-	CD161+	Foldchange	CD161-	CD161+	Foldchange	PD-1-	PD-1+	Foldchange	PD-1-	PD-1+	Foldchange
IFNγ	N	13675	16016	1.17	11551	13724	1.19	13724	16016	1.17	11551	13675	1.18
	T	4015	3388	0.84	5473	7957	1.45	7957	3388	0.43	5473	4015	0.73
TNFα	N	1313	2027	1.54	1114	1355	1.22	1355	2027	1.50	1114	1313	1.18
	T	1095	1371	1.25	1947	2458	1.26	2458	1371	0.56	1947	1095	0.56
IL-2	N	1238	1507	1.22	1363	1174	0.86	1174	1507	1.28	1363	1238	0.91
	T	987	1616	1.64	1445	1563	1.08	1563	1616	1.03	1445	987	0.68
GNLY	N	49.1	57.1	1.16	79.3	54.4	0.69	54.4	57.1	1.05	79.3	49.1	0.62
	T	40.6	39.9	0.98	75.2	42.1	0.56	42.1	39.9	0.95	75.2	40.6	0.54
PRF	N	48.7	66.4	1.36	68	75.6	1.11	75.6	66.4	0.88	68	48.7	0.72
	T	67	108	1.61	67	61.4	0.92	61.4	108	1.76	67	67	1.00
GZMB	N	584	600	1.03	556	516	0.93	516	600	1.16	556	584	1.05
	T	962	285	0.30	603	310	0.51	310	285	0.92	603	962	1.60

Supplementary Fig. 3 (a) IL-2, PRF, GZMB and TNF-α secretion level.

a

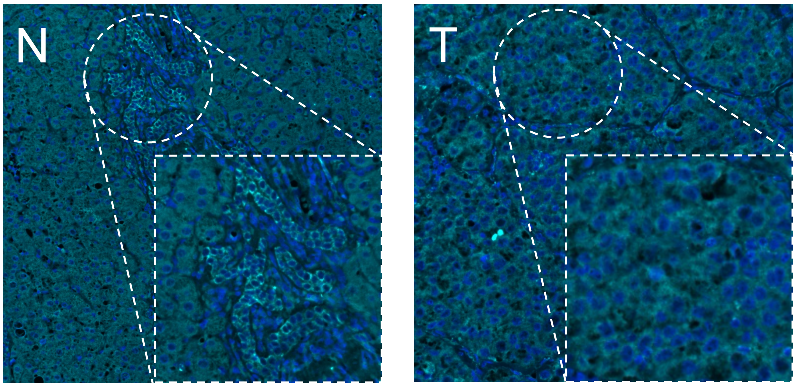

CD161 Stained by Opal520 dye

b

Cancer tissues displayed weak to moderate cytoplasmic positivity to CD161. Lymphomas were generally negative.

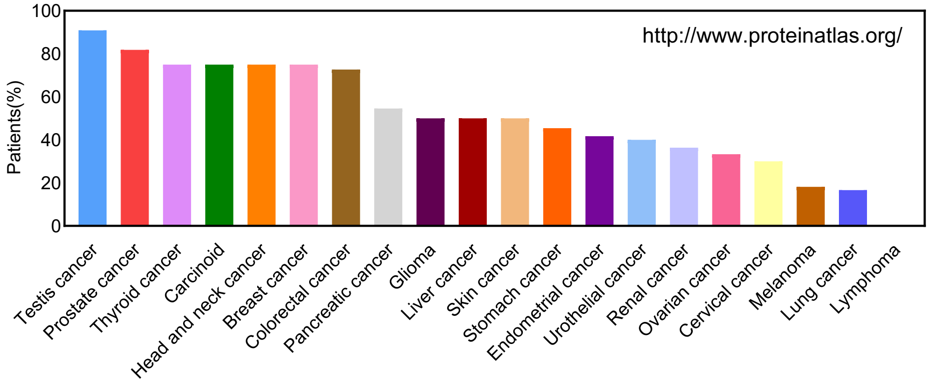

c

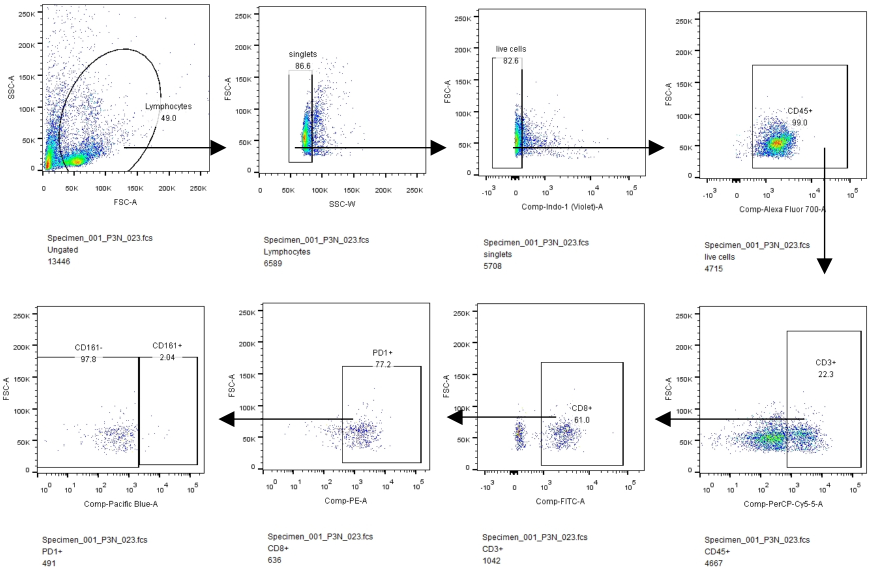

Supplementary Fig. 4 (a, b) Moderate CD161 expression in hepatocytes (some data from the protein atlas database). (c) Flow cytometry gating strategy.

a

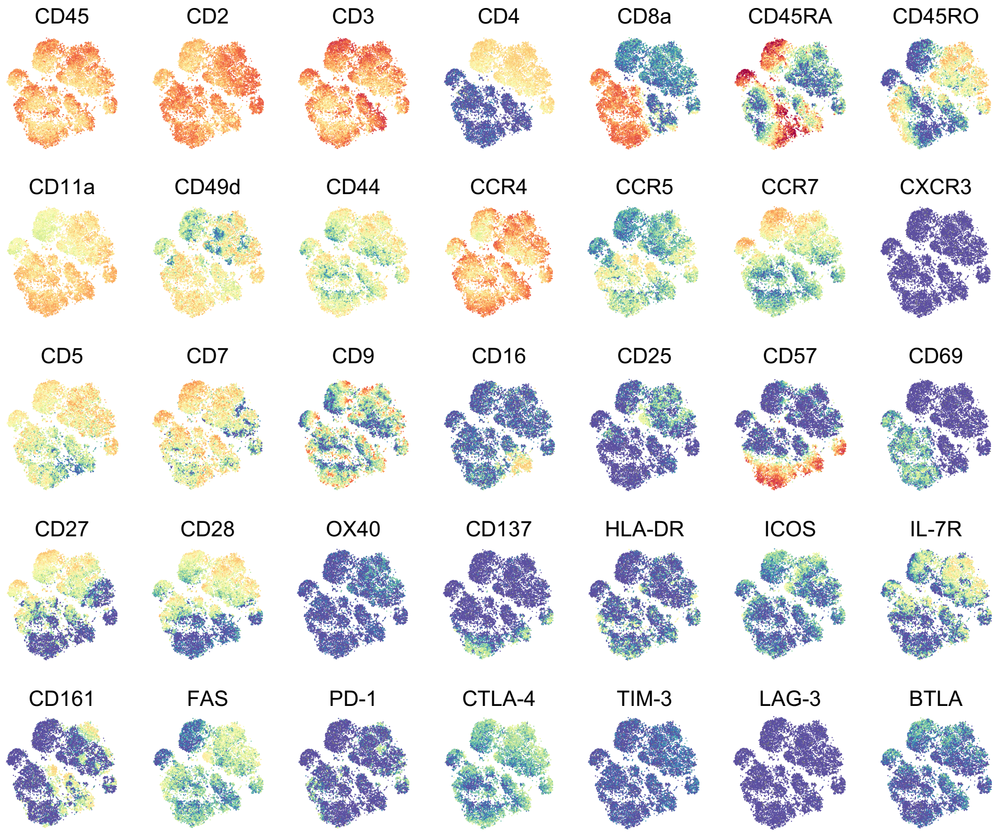

CD45
CD2
CD3
CD4
CD8a
CD45RA
CD45RO
CD11a
CD49d
CD44
CCR4
CCR5
CCR7
CXCR3
CD5
CD7
CD9
CD16
CD25
CD57
CD69
CD27
CD28
OX40
CD137
HLA-DR
ICOS
IL-7R
CD161
FAS
PD-1
CTLA-4
TIM-3
LAG-3
BTLA

Supplementary Fig. 5(a) tSNE plot of 35 markers.

Supplementary Table 1. Clinical Characteristics Of 20 HCC Patients

No.	Patient ID	Viral status	Grades	Sex	Race	Age	Tumor size(cm)	Tumor multiplicity	AFP level(ng/ml)	Sample type
1	209270	Hep B	III	M	China	46	4.6	1	4.8	Tisue
2	209283	Hep B	III	M	China	54	6.4	1	69.18	Tisue
3	205596	N	IV	M	China	55	9.9	2	11199	Tisue
4	205488	Hep B	III	M	China	50	7.3	1	3.6	Tisue
5	205516	N	III	M	China	69	3	1	169	Tisue
6	205832	N	III	M	China	66	7.2	1	9.9	Tisue
7	205854	Hep B	III	F	China	65	5.5	1	34.2	Tisue
8	205866	Hep B	III	F	China	71	3.2	1	9.9	Tisue
9	206112	Hep B	III	M	China	61	4	1	1210	Tisue
10	206169	Hep B	III	M	China	53	6.6	2	2.7	Tisue
11	206280	Hep B	III	M	China	70	4.8	1	91.8	Tisue
12	206988	Hep B	III	F	China	63	1.5	1	27.7	Tisue
13	206363	Hep B	III	M	China	56	10	3	2.7	Tisue
14	206548	Hep B	III	F	China	62	13.3	1	55840	Tisue
15	206579	Hep B	III	M	China	40	10	3	2989	Tisue
16	208041	N	III	F	China	70	5.5	1	210.2	Blood
17	208143	Hep B	III	M	China	47	8	3	170.9	Blood
18	208149	Hep B	III	M	China	37	8.9	1	299.3	Blood
19	208188	N	III	M	China	65	10	3	>1000	Blood
20	208336	Hep B	III	M	China	68	9	1	8	Blood

Supplementary Table 2. Chemical Reagents Used In CyTOF And Analysis-related Software/Algorithm			
Reagent name	Isotopes	Identifier	Source
Antibodies			
CD45	Y89	Cat# 3089003B	Fluidigm
CD45RA	169Tm	Cat# 3169008B	Fluidigm
CD45RO	165Ho	Cat# 3165011B	Fluidigm
CD49d	141Pr	Cat# 3141004B	Fluidigm
CD5	143Nd	Cat# 3143007B	Fluidigm
CD57	172Yb	Cat# 3172009B	Fluidigm
CD69	162Dy	Cat# 3162001B	Fluidigm
CD7	147Sm	Cat# 3147006B	Fluidigm
CD8a	146Nd	Cat# 3146001B	Fluidigm
CD9	171Yb	Cat# 3171009B	Fluidigm
CD95	152Sm	Cat# 3152017B	Fluidigm
HLA-DR	174Yb	Cat# 3174001B	Fluidigm
Tim-3	153Eu	Cat# 3153008B	Fluidigm
CD278	168Er	Cat# 3168024B	Fluidigm
CD11a	142Nd	Cat# 3142006B	Fluidigm
CD127	176Yb	Cat# 3176004B	Fluidigm
CD134	150Nd	Cat# 3150023B	Fluidigm
CD137	173Yb	Cat# 3173015B	Fluidigm
CD152	161Dy	Cat# 3161004B	Fluidigm
CD16	209Bi	Cat# 3209002B	Fluidigm
CD161	164Dy	Cat# 3164009B	Fluidigm
CD183	156Gd	Cat# 3156004B	Fluidigm
CD194	158Gd	Cat# 3158032A	Fluidigm
CD195	144Nd	Cat# 3144007A	Fluidigm
CD197	159Tb	Cat# 3159003A	Fluidigm
CD2	151Eu	Cat# 3151003B	Fluidigm
CD223	175Lu	Cat# 3175033B	Fluidigm
CD25	149Sm	Cat# 3149010B	Fluidigm
CD27	167Er	Cat# 3167002B	Fluidigm
CD272	163Dy	Cat# 3163009B	Fluidigm
CD279	155Gd	Cat# 3155009B	Fluidigm
CD28	160Gd	Cat# 3160003B	Fluidigm
CD3	170Er	Cat# 3170001B	Fluidigm
CD4	145Nd	Cat# 3140005B	Fluidigm
CD44	166Er	Cat# 3166001B	Fluidigm
Chemicals, Peptides, and Recombinant Proteins			
Cisplatin		Cat# 201192A	Fluidigm
Iridium		#422302	Fluidigm
Human TruStain FcX™		#422302	Biolegend
Software and Algorithms			
Cytobank		https://support.cytobank.org/hc/en-us/articles/206336147-FCS-file-concatenation-tool	
Concatenation tool			Cytobank
Cytofkit (tSNE, FlowSOM)		https://bioconductor.org/packages/release/bioc/html/destiny.html	Chen et al. (1)
Diffusion map			Cytobank

1. Chen H, Lau MC, Wong MT, Newell EW, Poidinger M, Chen J. Cytofkit: A Bioconductor Package for an Integrated Mass Cytometry Data Analysis Pipeline. PLoS Comput Biol 2016;12:e1005112.

Supplementary Table 3. Mean Expression of 35 Markers (tissue)

Clusters	CD45	CD49d	CD11a	CD5	CD195	CD4	CD8a	CD7	CD25	CD134	CD2	CD95	TIM_3	CD279	CD183	CD194	CD197
T01	0.57	0.33	0.47	0.14	0.35	0.03	0.79	0.64	0.03	0.02	0.70	0.31	0.02	0.03	0.18	0.07	0.01
T02	0.46	0.33	0.39	0.34	0.26	0.02	0.69	0.30	0.01	0.01	0.65	0.25	0.01	0.08	0.08	0.05	0.02
T03	0.59	0.57	0.46	0.42	0.17	0.04	0.81	0.23	0.00	0.01	0.72	0.26	0.02	0.08	0.11	0.11	0.01
T04	0.63	0.37	0.49	0.44	0.36	0.04	0.79	0.43	0.01	0.01	0.76	0.39	0.03	0.26	0.25	0.14	0.03
T05	0.37	0.12	0.16	0.12	0.12	0.06	0.32	0.16	0.02	0.02	0.33	0.12	0.01	0.19	0.22	0.19	0.02
T06	0.60	0.53	0.28	0.08	0.12	0.16	0.14	0.07	0.05	0.01	0.20	0.24	0.04	0.16	0.26	0.23	0.10
T07	0.59	0.24	0.23	0.50	0.06	0.45	0.09	0.44	0.08	0.01	0.62	0.14	0.03	0.09	0.15	0.14	0.41
T08	0.57	0.31	0.40	0.47	0.28	0.50	0.10	0.43	0.45	0.12	0.72	0.58	0.05	0.21	0.21	0.22	0.04
T09	0.68	0.19	0.37	0.44	0.06	0.02	0.48	0.71	0.09	0.01	0.71	0.21	0.03	0.11	0.48	0.14	0.03
T10	0.63	0.37	0.49	0.53	0.29	0.54	0.12	0.26	0.08	0.04	0.77	0.51	0.04	0.47	0.30	0.17	0.06
T11	0.70	0.30	0.49	0.45	0.48	0.12	0.75	0.60	0.06	0.03	0.84	0.43	0.05	0.39	0.39	0.39	0.04
T12	0.63	0.34	0.46	0.53	0.39	0.56	0.14	0.27	0.09	0.02	0.82	0.45	0.05	0.20	0.25	0.15	0.06
T13	0.64	0.32	0.48	0.40	0.37	0.04	0.77	0.44	0.01	0.02	0.75	0.35	0.03	0.44	0.22	0.13	0.01
T14	0.60	0.32	0.43	0.23	0.35	0.03	0.55	0.49	0.01	0.01	0.65	0.28	0.02	0.10	0.14	0.10	0.01
T15	0.65	0.30	0.49	0.43	0.36	0.05	0.78	0.43	0.01	0.02	0.74	0.46	0.05	0.46	0.25	0.13	0.02
T16	0.57	0.25	0.24	0.49	0.06	0.04	0.78	0.51	0.01	0.01	0.63	0.06	0.01	0.09	0.22	0.15	0.40
T17	0.58	0.24	0.42	0.36	0.44	0.04	0.30	0.48	0.02	0.02	0.05	0.28	0.00	0.11	0.16	0.12	0.02
T18	0.64	0.43	0.48	0.41	0.31	0.21	0.56	0.43	0.06	0.03	0.74	0.40	0.05	0.23	0.24	0.22	0.04
T19	0.62	0.58	0.48	0.51	0.23	0.52	0.13	0.10	0.00	0.01	0.78	0.32	0.04	0.18	0.14	0.11	0.03
T20	0.67	0.32	0.48	0.39	0.24	0.05	0.82	0.51	0.03	0.01	0.75	0.47	0.07	0.61	0.33	0.17	0.02
T21	0.57	0.24	0.41	0.38	0.45	0.03	0.68	0.45	0.01	0.01	0.76	0.27	0.03	0.08	0.10	0.06	0.01
T22	0.61	0.41	0.46	0.03	0.22	0.02	0.13	0.56	0.01	0.01	0.62	0.17	0.03	0.02	0.08	0.06	0.01
T23	0.23	0.18	0.09	0.02	0.06	0.05	0.06	0.04	0.01	0.01	0.08	0.22	0.01	0.03	0.18	0.07	0.07
T24	0.45	0.35	0.35	0.42	0.20	0.45	0.07	0.20	0.04	0.01	0.68	0.32	0.02	0.07	0.11	0.07	0.07
T25	0.51	0.50	0.16	0.06	0.11	0.16	0.15	0.08	0.06	0.01	0.23	0.21	0.03	0.11	0.20	0.19	0.03
T26	0.56	0.26	0.42	0.29	0.37	0.02	0.09	0.44	0.02	0.01	0.69	0.29	0.03	0.11	0.15	0.09	0.02
T27	0.57	0.33	0.44	0.02	0.18	0.02	0.11	0.61	0.03	0.00	0.05	0.12	0.02	0.03	0.07	0.06	0.00
T28	0.65	0.41	0.44	0.54	0.22	0.57	0.13	0.36	0.12	0.02	0.80	0.43	0.04	0.14	0.34	0.20	0.14
T29	0.48	0.16	0.25	0.05	0.04	0.03	0.09	0.05	0.01	0.01	0.07	0.17	0.00	0.07	0.10	0.12	0.01
T30	0.62	0.47	0.45	0.22	0.20	0.01	0.19	0.27	0.00	0.01	0.64	0.16	0.02	0.02	0.05	0.04	0.01

Clusters	CD28	CD152	CD69	CD272	CD161	CD45RO	CD44	CD27	CD278	CD45RA	CD3	CD9	CD57	CD137	HLA_DR	CD223	CD127	CD16
T01	0.06	0.02	0.66	0.12	0.03	0.12	0.07	0.01	0.02	0.09	0.44	0.21	0.10	0.01	0.03	0.00	0.04	0.02
T02	0.13	0.00	0.36	0.02	0.02	0.07	0.03	0.03	0.03	0.02	0.36	0.02	0.02	0.00	0.02	0.00	0.05	0.02
T03	0.10	0.00	0.27	0.01	0.04	0.12	0.09	0.01	0.06	0.18	0.51	0.07	0.67	0.15	0.06	0.00	0.02	0.04
T04	0.27	0.01	0.56	0.06	0.03	0.25	0.09	0.09	0.11	0.03	0.51	0.05	0.02	0.01	0.06	0.00	0.08	0.02
T05	0.20	0.01	0.28	0.03	0.10	0.13	0.04	0.03	0.05	0.11	0.29	0.10	0.05	0.01	0.10	0.00	0.04	0.16
T06	0.22	0.01	0.25	0.06	0.03	0.18	0.15	0.06	0.06	0.36	0.22	0.16	0.04	0.01	0.43	0.01	0.04	0.08
T07	0.35	0.03	0.22	0.04	0.02	0.05	0.09	0.25	0.11	0.30	0.63	0.06	0.03	0.00	0.03	0.00	0.24	0.04
T08	0.42	0.04	0.40	0.04	0.03	0.34	0.11	0.20	0.56	0.02	0.53	0.04	0.01	0.04	0.06	0.00	0.04	0.02
T09	0.12	0.05	0.74	0.20	0.03	0.12	0.09	0.31	0.06	0.34	0.52	0.16	0.02	0.00	0.02	0.00	0.06	0.01
T10	0.39	0.05	0.54	0.09	0.12	0.35	0.11	0.13	0.37	0.02	0.56	0.05	0.08	0.01	0.07	0.00	0.03	0.02
T11	0.56	0.14	0.86	0.33	0.66	0.25	0.07	0.11	0.17	0.03	0.66	0.14	0.03	0.01	0.05	0.00	0.50	0.05
T12	0.37	0.07	0.61	0.11	0.47	0.30	0.13	0.05	0.19	0.02	0.54	0.05	0.01	0.00	0.03	0.00	0.36	0.02
T13	0.25	0.03	0.66	0.12	0.50	0.27	0.08	0.10	0.15	0.04	0.51	0.04	0.02	0.01	0.05	0.00	0.02	0.04
T14	0.11	0.02	0.55	0.08	0.34	0.04	0.06	0.03	0.02	0.37	0.58	0.04	0.05	0.01	0.05	0.00	0.02	0.04
T15	0.26	0.02	0.64	0.11	0.07	0.39	0.11	0.18	0.26	0.04	0.53	0.06	0.53	0.06	0.09	0.00	0.01	0.03
T16	0.29	0.00	0.30	0.04	0.01	0.04	0.06	0.25	0.07	0.45	0.54	0.11	0.01	0.00	0.03	0.00	0.25	0.05
T17	0.34	0.04	0.60	0.13	0.59	0.22	0.08	0.05	0.04	0.09	0.58	0.06	0.01	0.00	0.03	0.00	0.29	0.02
T18	0.32	0.04	0.52	0.09	0.25	0.28	0.13	0.08	0.13	0.26	0.54	0.10	0.25	0.06	0.09	0.00	0.19	0.73
T19	0.14	0.02	0.13	0.01	0.07	0.28	0.14	0.01	0.11	0.06	0.53	0.08	0.63	0.15	0.06	0.00	0.09	0.02
T20	0.30	0.03	0.69	0.13	0.04	0.39	0.07	0.29	0.44	0.03	0.51	0.07	0.03	0.04	0.10	0.01	0.01	0.03
T21	0.26	0.03	0.61	0.11	0.53	0.17	0.05	0.03	0.04	0.06	0.50	0.07	0.01	0.00	0.02	0.00	0.27	0.02
T22	0.06	0.01	0.41	0.06	0.44	0.07	0.04	0.03	0.01	0.62	0.05	0.04	0.09	0.02	0.05	0.00	0.01	0.45
T23	0.10	0.01	0.07	0.01	0.02	0.17	0.03	0.02	0.04	0.23	0.06	0.29	0.02	0.01	0.09	0.00	0.01	0.07
T24	0.23	0.03	0.25	0.02	0.06	0.11	0.05	0.02	0.07	0.01	0.38	0.03	0.01	0.00	0.02	0.00	0.19	0.02
T25	0.18	0.02	0.38	0.05	0.05	0.22	0.27	0.07	0.04	0.15	0.18	0.83	0.32	0.02	0.15	0.01	0.04	0.11
T26	0.24	0.02	0.52	0.09	0.32	0.17	0.06	0.05	0.05	0.05	0.54	0.06	0.04	0.00	0.03	0.00	0.15	0.02
T27	0.07	0.02	0.49	0.08	0.47	0.05	0.04	0.03	0.01	0.56	0.05	0.02	0.04	0.00	0.02	0.00	0.01	0.28
T28	0.42	0.05	0.37	0.05	0.10	0.32	0.18	0.09	0.13	0.04	0.55	0.06	0.01	0.00	0.04	0.00	0.43	0.03
T29	0.12	0.00	0.05	0.01	0.03	0.28	0.14	0.01	0.01	0.37	0.06	0.04	0.01	0.00	0.03	0.00	0.03	1.00
T30	0.03	0.00	0.22	0.02	0.20	0.05	0.13	0.02	0.07	0.39	0.69	0.07	0.47	0.08	0.03	0.00	0.04	0.26

Normalized intensity

Supplementary Table 4. The abundance of 30 Clusters in tumor tissues

Clusters	205488N	205488T	205516N	205516T	205596N	205596T	205832N	205832T	205854N	205854T	205866N	205866T	206112N	206112T	206169N	206169T	206280N	206280T	206363N	206363T	206548N	206548T	206579N	206579T	206988N	206988T	209270N	209270T	209283N	209283T
T01	0.78	0.38	0.76	0.64	4.46	0.22	3.82	0.32	0.1	0.28	0.86	0.74	0.28	0.04	0.56	0.18	0.48	2.72	0.08	0.14	1.84	0.14	4.42	1.44	2.02	0.5	0.14	0	0.1	0.06
T02	16.54	7.68	18.76	11.86	17.96	6.06	19.5	2.26	8.42	4.58	15.96	3.48	3.4	0.7	3.56	0.48	2.06	1.3	0.06	0.92	5.88	3.22	4.94	6.7	1.46	2.08	1.72	0.06	2.02	0.62
T03	1.46	1.04	5.9	1.14	4.76	4.54	5.78	5.46	7.5	3.92	0.88	5.08	3.08	5.3	1.88	1.98	4.32	5.58	1.26	1.94	0.54	0.28	1.48	0.9	1.12	0.18	0.46	0.1	0.1	0.62
T04	6.76	8.04	17.56	19.8	8.68	3.52	26.34	17.68	11.5	11.48	10.8	18.54	8.74	2.5	12.04	8.06	10.66	9.16	15.82	16.92	22.1	6.52	27.42	19.66	12.06	8.06	12.04	0.52	2.3	4.3
T05	2.02	1.4	1.16	4.44	0.72	0.66	0.74	1.52	1.44	1.46	2.76	1.06	0.98	0.1	4.66	2.18	19.78	1.02	0.74	0.3	0.68	1.82	0.08	1.14	0.4	4.58	0.24	0.36	8.94	0.2
T06	0.36	0.92	0.32	1.06	0.38	0.34	0.12	0.46	0.82	1.08	0.22	1.96	0.52	0.1	3	3.8	6.26	1.04	0.26	0.1	0.56	0.44	0.74	0.4	0.16	3.26	0.14	0.2	1.76	0.34
T07	0.7	0.08	1.42	0.28	1.08	0.22	0.82	1.3	0.76	0.16	0.06	0.92	1.7	0.88	1.16	0.32	1.64	0.4	4	0.96	0.76	0.7	1.96	1.26	0.2	0.08	0.6	0	0.88	0.7
T08	0.94	9.94	2.04	11.56	1.8	22.56	0.96	9.58	1.42	10.48	0.52	8.14	5.68	23.06	2.54	15.88	1.72	13.48	1.12	4.32	2.6	29.06	3.44	16.32	0.72	7.98	0.44	1.8	0.36	2.36
T09	0.02	0.16	0	0.04	0	0	0.04	0.06	0.06	0.06	0.02	0.02	0.08	0.02	0.08	0.36	0.32	7.94	0.02	0	0.08	0.32	0.24	0.24	0.2	0.94	0.12	0.02	0	0.04
T10	1.28	2.98	3.62	11.04	1.34	1.4	2.5	5.4	2	13.9	1.14	5.74	3.14	6.3	3.8	7.98	2.62	10.8	2.9	3.36	4.42	12.82	2.92	6.34	2.94	8.1	1.58	0.2	0.48	2.48
T11	0.22	0.38	0.16	0.62	0.12	0.32	0.04	0.44	0.14	0.16	0.04	0.9	0.04	0.04	2.34	4.72	1.34	0.06	15.02	12.4	0.02	0.16	0.2	0.2	0.24	0.32	0	0	0.02	0
T12	6.82	14.38	2.58	3.26	2.28	8.34	2.42	3.78	9.64	15.9	1.6	3.26	8.76	20.64	5.3	7.62	3.18	9.18	10.6	17.76	7.54	6.64	3.8	5.16	9.5	4.38	3.28	0.22	6.18	12.14
T13	4.9	1.42	2.04	1.44	1.28	0.18	1.12	0.54	8.3	5.3	6.02	0.82	1.74	0.26	4.7	1.54	5.42	2.04	13.38	4.7	5.32	0.18	21.12	4.98	3.2	1.14	8.28	0.06	3.66	2.92
T14	4.72	0.5	13.86	0.52	8.36	0.38	4.32	0.5	13.28	1.12	12.14	0.96	4.86	1.12	2.78	0.36	5.98	1.46	4.4	0.28	7.68	0.2	2.78	0.84	7.54	0.3	4.1	0.12	4.36	2.86
T15	0.6	1.02	1.1	2.6	0.46	0.36	0.28	2.84	0.36	0.56	0.22	2.08	2.7	0.4	3.24	2.2	2.6	4.24	0.28	0.5	9.64	3.3	1.28	0.5	4.58	6.46	0.1	0	0.04	0.06
T16	0.34	0.04	0.26	0.12	0.22	0	0.38	0.36	0.3	0.04	0	0.24	0.92	0.24	0.88	0.14	0.76	0.12	1.94	0.52	0.3	0.08	1.16	0.56	0.22	0.16	0.34	0	0.04	0.06
T17	1.3	0.08	0.02	0	0.08	0.02	0.22	0	0.08	0.08	0.02	0.02	0.32	0.02	0.64	0.14	0.14	0	0.06	0.16	0.08	0.02	0.26	0.48	1.66	0.1	0.06	0	0.14	0.12
T18	0.28	0.3	0.16	0.28	0.96	0.72	0.18	0.36	0.48	0.18	0.02	0.6	0.46	0.56	1.38	0.8	1.14	0	3.94	0.36	1.76	0.52	2.32	1.58	1.06	0.06	0.12	0.08	1.76	0.2
T19	0.92	0.64	2.24	0.66	0.5	0.6	1.18	1.9	2.16	1.4	0.36	2.22	1	1.86	0.64	0.58	0.38	0.68	0.2	0.44	0.62	0.42	0.42	0.12	0.74	0.12	0.16	0.02	0.24	1.96
T20	0	0.2	0.14	2.2	0.06	0.3	0.26	16.4	0.4	1.24	0.04	12.5	0.42	1.9	2.18	7.42	9.24	18.52	8.86	1.36	1.28	15.8	0.64	5.52	0.72	16.96	0.22	0.8	0.02	0.06
T21	18.58	3.7	7.06	1.84	7.88	3.04	7.16	0.7	6.92	2.2	26.26	2.1	19.46	5.7	8.28	2.22	2.46	0.28	0.94	8.82	11.88	1.66	6.58	6.76	25.14	0.94	7.78	0.02	23.04	7.18
T22	0.12	0.1	0.08	0.02	0.2	0.04	0.26	0.12	0.14	0.1	0.16	0.34	0.48	0.04	0.94	0.58	1.76	0.2	0.06	0.02	0.22	0	0.46	0.06	0.56	0.5	36.62	3.26	6.4	3.68
T23	0.06	0.9	0.04	1.28	0	0.44	0.04	0.1	0.06	0.16	0.1	0.12	0.22	0.08	0.8	0.34	1.56	0.02	0.02	0.06	0.12	0.42	0	0.36	0.16	1.56	0.86	89.88	6.56	0.46
T24	12.84	32.86	8.72	12.88	22.66	37	6.12	4.86	11.66	13.54	4.58	7.16	7.02	3.28	7.48	3.52	2.32	2.18	0.2	2.7	2.86	6.94	2.46	8.52	1.44	9.78	1.08	0.48	5.94	5.48
T25	0.02	0.02	0.08	0.02	0	0.02	0	0.02	0.06	0	0.02	0.08	0.04	0.02	0.04	0	0.9	0	0.18	0.34	0.32	0.02	0.02	0.02	0	0.04	0.38	0.86	0.44	0.06
T26	13.82	5.8	5.04	4.44	8.5	3.64	12.78	2.06	3.88	1.72	14.14	2.24	7.58	2.94	5.52	3.24	2.3	1.24	3.12	4.88	6.9	1.24	2.94	3.82	9.88	1.78	4.24	0.1	2.14	2.76
T27	0.08	0.16	0.08	0.02	0.06	0	0	0	0.12	0.04	0.12	0.02	0.06	0	0.3	0.16	0.48	0.04	0	0	0.04	0	0	0.02	0.18	0.2	5.32	0.26	3.96	2.78
T28	2	4.14	4.02	5.5	1.54	3.64	2.28	20.82	7.6	8.52	0.58	18.54	10.8	4.62	18.14	22.82	7	5.92	10.34	15.64	2.7	6.04	4.66	5.68	10.82	19.26	5.08	0.36	2.14	34.2
T29	0.02	0	0.04	0	0.04	0.1	0.12	0.04	0.1	0.06	0.02	0.04	0.1	0.02	0.72	0.22	0.94	0	0.16	0.02	0.06	0.02	0.24	0.12	0.16	0.04	4.16	0.18	13.92	5.9
T30	1.5	0.74	0.74	0.44	3.62	1.34	0.22	0.12	0.3	0.28	0.34	0.08	5.42	17.26	0.42	0.16	0.24	0.38	0.04	0.08	1.2	1.02	1.02	0.3	0.92	0.14	0.34	0.04	2.06	5.4

Cell percentage(%)

Supplementary Table 5. the difference of Gene expression between CD8+PD-1+CD161- and CD8+PD-1+

Gene	Pvalue	Log2Foldchange	PCT1	PCT2	AdjustPvalue	Cluster
CCL5	4.16E-44	1.084818138	0.996	0.914	5.06E-40	CD161-
GZMB	5.36E-35	1.733726823	0.704	0.133	6.53E-31	CD161-
NKG7	4.50E-33	1.05234195	0.949	0.648	5.48E-29	CD161-
GZMA	1.49E-27	1.125122954	0.863	0.41	1.81E-23	CD161-
HLA-DRB5	7.53E-26	1.137920364	0.805	0.319	9.18E-22	CD161-
COTL1	3.73E-22	0.926443276	0.736	0.262	4.55E-18	CD161-
CD74	5.79E-21	0.775220671	0.939	0.638	7.06E-17	CD161-
HLA-DRA	2.82E-18	1.458540835	0.39	0.043	3.44E-14	CD161-
CD52	1.24E-17	0.757042732	0.787	0.371	1.51E-13	CD161-
GZMH	1.31E-17	0.86851672	0.704	0.314	1.60E-13	CD161-
ACTB	4.44E-17	0.507157713	0.996	0.971	5.41E-13	CD161-
CXCL13	1.44E-15	1.644955847	0.303	0.019	1.76E-11	CD161-
GAPDH	1.70E-14	0.45838998	0.989	0.938	2.07E-10	CD161-
RP11-291B21.2	4.42E-14	1.222690419	0.325	0.043	5.38E-10	CD161-
MIR4435-1HG	5.99E-14	0.991082627	0.321	0.038	7.30E-10	CD161-
SH3BGRL3	1.58E-13	0.434317024	0.993	0.848	1.92E-09	CD161-
PFN1	1.54E-12	0.484609723	0.935	0.819	1.87E-08	CD161-
HLA-DRB1	4.20E-12	0.886513614	0.419	0.119	5.11E-08	CD161-
TIGIT	1.87E-11	0.930326709	0.329	0.067	2.27E-07	CD161-
PRF1	3.20E-11	0.849910154	0.437	0.152	3.89E-07	CD161-
GNLY	3.79E-11	1.844236841	0.282	0.048	4.61E-07	CD161-
PARK7	1.27E-10	0.672585131	0.527	0.2	1.55E-06	CD161-
HLA-DPB1	4.53E-10	0.573662168	0.57	0.233	5.51E-06	CD161-
DUSP4	6.15E-10	0.689960029	0.473	0.176	7.49E-06	CD161-
TMSB10	7.14E-10	0.279043605	0.996	0.995	8.69E-06	CD161-
HLA-DPA1	9.38E-10	0.523176896	0.686	0.333	1.14E-05	CD161-
RARRES3	1.10E-09	0.630736666	0.646	0.367	1.34E-05	CD161-
HLA-DQA2	1.14E-09	0.594706882	0.513	0.195	1.38E-05	CD161-
FKBP1A	1.24E-09	0.778913718	0.321	0.081	1.51E-05	CD161-
C12orf75	1.46E-09	0.763448068	0.379	0.119	1.77E-05	CD161-
CD7	2.04E-09	0.484095347	0.794	0.586	2.48E-05	CD161-
ATP5G3	3.59E-09	0.576111925	0.469	0.176	4.37E-05	CD161-
PSMB9	3.69E-09	0.631328557	0.563	0.271	4.49E-05	CD161-
CCL3	4.24E-09	1.18623866	0.264	0.057	5.17E-05	CD161-
LAG3	7.37E-09	0.788307535	0.401	0.148	8.98E-05	CD161-
YWHAB	1.01E-08	0.571544425	0.592	0.286	0.000122712	CD161-
PSMB10	1.29E-08	0.78364701	0.296	0.081	0.000156627	CD161-
PTPRCAP	2.12E-08	0.438387259	0.91	0.776	0.000258753	CD161-
SEC61G	2.13E-08	0.610207498	0.487	0.205	0.000259976	CD161-
CD27	3.44E-08	0.714538696	0.253	0.057	0.00041847	CD161-
HMGB2	4.20E-08	0.558681633	0.329	0.1	0.000511103	CD161-
LGALS3	7.34E-08	0.684699719	0.408	0.167	0.000893809	CD161-
ATP5J2	7.40E-08	0.480727102	0.552	0.276	0.00090098	CD161-
CTSD	7.55E-08	0.721110052	0.278	0.076	0.000919709	CD161-
FABP5	1.23E-07	0.639895556	0.39	0.157	0.001496405	CD161-
RHOA	1.23E-07	0.506674761	0.588	0.319	0.00149926	CD161-
SERF2	1.76E-07	0.276063837	0.921	0.829	0.002143593	CD161-
BLOC1S1	1.96E-07	0.541502989	0.354	0.124	0.002391384	CD161-
HLA-DQA1	2.31E-07	0.556986293	0.26	0.067	0.002812767	CD161-
SHFM1	2.80E-07	0.479284936	0.361	0.133	0.003416145	CD161-
LSP1	3.08E-07	0.575769431	0.44	0.2	0.003755198	CD161-
PPIB	3.34E-07	0.444480404	0.603	0.333	0.004064102	CD161-
IL32	3.42E-07	0.35152753	0.913	0.71	0.00416764	CD161-
CD3G	9.40E-07	0.577706242	0.285	0.095	0.011455019	CD161-
COX6B1	9.75E-07	0.406650747	0.588	0.333	0.011877073	CD161-
NDUFA1	1.05E-06	0.43492835	0.599	0.324	0.012789521	CD161-
RAC2	1.27E-06	0.402858047	0.729	0.467	0.015437269	CD161-
CD3D	1.29E-06	0.369310923	0.805	0.605	0.015674021	CD161-
LSM7	1.39E-06	0.43478165	0.404	0.171	0.016966421	CD161-
ARPC1B	1.51E-06	0.565117645	0.451	0.229	0.018371204	CD161-
LINC00152	1.55E-06	0.521073702	0.44	0.205	0.018836209	CD161-
TPI1	1.82E-06	0.542277233	0.563	0.343	0.022220856	CD161-
KLRD1	1.98E-06	0.564801659	0.282	0.095	0.024090381	CD161-
HERPUD1	2.52E-06	0.549064546	0.256	0.081	0.030644081	CD161-
CLIC1	3.16E-06	0.325683734	0.805	0.571	0.038518567	CD161-
RABAC1	4.72E-06	0.380703923	0.574	0.324	0.057534163	CD161-
CAPZB	4.91E-06	0.494674807	0.448	0.224	0.059796611	CD161-
IFI27L2	6.03E-06	0.492406354	0.303	0.114	0.073504437	CD161-
CASP4	6.90E-06	0.55449312	0.274	0.1	0.084091472	CD161-
CLEC2B	8.57E-06	0.347716989	0.549	0.295	0.104340144	CD161-
LY6E	1.08E-05	0.444681192	0.415	0.21	0.130979571	CD161-
PPP1CA	1.25E-05	0.462750086	0.314	0.129	0.152720392	CD161-
CTSC	1.37E-05	0.584492992	0.343	0.152	0.166696334	CD161-
TCEB2	1.46E-05	0.354097197	0.704	0.448	0.178069836	CD161-
UBE2L3	1.55E-05	0.445612654	0.256	0.09	0.188750058	CD161-
UCP2	1.65E-05	0.390437229	0.318	0.129	0.200709188	CD161-
C8orf59	1.93E-05	0.423950985	0.357	0.157	0.23543584	CD161-
ALDOA	2.75E-05	0.347846798	0.585	0.367	0.335433084	CD161-
RPS26	2.85E-05	0.251225395	0.852	0.705	0.34752485	CD161-
TRAPPC1	2.95E-05	0.337170434	0.303	0.124	0.358824751	CD161-
GSTP1	3.43E-05	0.250347253	0.534	0.29	0.418176233	CD161-
NDUFA12	3.88E-05	0.430406917	0.271	0.11	0.472491233	CD161-
NDUFB1	4.60E-05	0.449365334	0.3	0.133	0.560862464	CD161-
PSMB3	4.89E-05	0.460346317	0.314	0.143	0.595444429	CD161-
LDHB	4.95E-05	0.335065021	0.386	0.181	0.60315792	CD161-
APOBEC3G	5.31E-05	0.527891441	0.278	0.124	0.647098786	CD161-
CXCR6	5.59E-05	0.403883457	0.39	0.2	0.680253019	CD161-
ARPC3	6.14E-05	0.285436031	0.671	0.433	0.747308511	CD161-
EIF3F	6.28E-05	0.341008868	0.325	0.148	0.765211393	CD161-
PSME2	6.48E-05	0.355029055	0.581	0.362	0.789797501	CD161-
COX8A	7.20E-05	0.325502593	0.592	0.362	0.877464829	CD161-
EVL	7.59E-05	0.402235179	0.433	0.224	0.924087819	CD161-
RAC1	8.72E-05	0.372776579	0.336	0.157	1	CD161-
PSME1	9.68E-05	0.275301704	0.787	0.581	1	CD161-
CORO1A	0.000116581	0.397208444	0.458	0.267	1	CD161-
HCST	0.00012185	0.260219982	0.78	0.581	1	CD161-
CSNK2B	0.000138514	0.3977886	0.35	0.176	1	CD161-
ATP5G2	0.000172694	0.26892776	0.812	0.681	1	CD161-
SSR2	0.000173653	0.362577326	0.458	0.267	1	CD161-
LCK	0.000175757	0.308175039	0.347	0.162	1	CD161-
ATP5D	0.000180654	0.25968011	0.469	0.262	1	CD161-
UBE2D2	0.000183446	0.327811549	0.397	0.214	1	CD161-
TMEM258	0.000185059	0.356822124	0.422	0.233	1	CD161-
CTSW	0.000195828	0.380804456	0.708	0.543	1	CD161-
HIGD2A	0.00028572	0.250089478	0.614	0.381	1	CD161-
WDR83OS	0.000319689	0.316255926	0.397	0.214	1	CD161-
DYNLL1	0.000330557	0.397334948	0.253	0.114	1	CD161-
CFL1	0.000413029	0.326787673	0.765	0.571	1	CD161-
IL2RG	0.000414707	0.277596752	0.581	0.362	1	CD161-
ACTG1	0.000424062	0.277647325	0.758	0.595	1	CD161-
H2AFV	0.00045595	0.313775694	0.289	0.143	1	CD161-
PAIP2	0.00046656	0.27071327	0.271	0.124	1	CD161-
ARPC2	0.000470838	0.289724806	0.599	0.405	1	CD161-
NEDD8	0.000472401	0.25637867	0.274	0.124	1	CD161-
PGK1	0.000557759	0.294163392	0.422	0.243	1	CD161-
CARD16	0.000598698	0.321489449	0.274	0.129	1	CD161-
ENO1	0.000612063	0.387167682	0.285	0.143	1	CD161-
CKLF	0.000652875	0.328499252	0.43	0.248	1	CD161-
ETS1	0.000692003	0.349822028	0.3	0.152	1	CD161-
APRT	0.000737215	0.30752978	0.354	0.19	1	CD161-
TMBIM6	0.000741166	0.295830335	0.523	0.329	1	CD161-
CALM3	0.000750021	0.267057319	0.282	0.138	1	CD161-
SRP14	0.000770709	0.295823358	0.639	0.443	1	CD161-
C9orf16	0.000836629	0.328593356	0.422	0.257	1	CD161-
PSMB2	0.000851421	0.29980909	0.271	0.129	1	CD161-
AC092580.4	0.00100078	0.342851552	0.527	0.357	1	CD161-
RPL23	0.001043401	0.269529354	0.448	0.267	1	CD161-
TOMM5	0.001161067	0.281489504	0.415	0.238	1	CD161-
MRPS6	0.001239977	0.307774879	0.321	0.176	1	CD161-
CMC2	0.001245481	0.256392157	0.329	0.167	1	CD161-
CDK2AP2	0.001270326	0.301992272	0.264	0.133	1	CD161-
ARF5	0.001522832	0.30148694	0.264	0.133	1	CD161-
USMG5	0.001710498	0.262919847	0.538	0.352	1	CD161-
DYNLT1	0.001769536	0.294855576	0.253	0.124	1	CD161-
ATP5C1	0.001958264	0.344056483	0.26	0.133	1	CD161-
SUB1	0.001968422	0.265959577	0.733	0.538	1	CD161-
COX6C	0.001983146	0.292109848	0.643	0.452	1	CD161-
COX6A1	0.00221707	0.264254998	0.693	0.495	1	CD161-
UQCRH	0.002262692	0.267798823	0.394	0.243	1	CD161-
LITAF	0.002276249	0.291936251	0.43	0.262	1	CD161-
KRTCAP2	0.002338693	0.267174795	0.458	0.286	1	CD161-
NME1-NME2	0.002834312	0.25074724	0.451	0.295	1	CD161-
RGS10	0.002846995	0.282114656	0.433	0.281	1	CD161-
HLA-F	0.003023856	0.299017287	0.289	0.162	1	CD161-
EIF3H	0.003261762	0.298605048	0.307	0.176	1	CD161-
SAMSN1	0.003351021	0.273390471	0.26	0.138	1	CD161-
YBX1	0.003798201	0.289736307	0.462	0.305	1	CD161-
SLC25A6	0.004166129	0.275155612	0.52	0.362	1	CD161-
GZMK	0.005262785	0.386687853	0.52	0.41	1	CD161-
LGALS1	0.00734395	0.408673142	0.354	0.233	1	CD161-
DAZAP2	0.00883745	0.252974077	0.332	0.205	1	CD161-
DUSP2	4.67E-22	0.902890623	0.871	0.653	5.69E-18	CD161+
RPS12	1.91E-19	0.334329577	1	1	2.33E-15	CD161+
EEF1A1	7.45E-16	0.415236823	0.995	0.964	9.08E-12	CD161+
ZFP36	1.35E-15	0.84193373	0.786	0.603	1.65E-11	CD161+
RPL30	4.89E-15	0.288125808	1	1	5.95E-11	CD161+
RPS27A	2.32E-13	0.297240245	0.99	0.996	2.83E-09	CD161+
PDCD1	6.16E-13	0.254235253	1	1	7.51E-09	CD161+
MALAT1	8.07E-13	0.551747099	0.976	0.953	9.83E-09	CD161+
RPL32	1.44E-11	0.283049092	1	1	1.76E-07	CD161+
ZFP36L2	2.65E-10	0.673391587	0.852	0.798	3.23E-06	CD161+
RPL34	3.04E-10	0.263568783	1	0.993	3.70E-06	CD161+
KLRB1	1.58E-09	0.896850068	0.381	0.152	1.92E-05	CD161+
RPS10	3.86E-09	0.269231306	0.99	0.982	4.70E-05	CD161+
RPS14	5.56E-09	0.268362275	0.99	0.989	6.77E-05	CD161+
RPL9	9.67E-09	0.396924363	0.814	0.751	0.000117834	CD161+
FAM177A1	1.45E-08	0.72914498	0.567	0.401	0.000176407	CD161+
IL7R	2.56E-08	0.969744744	0.252	0.076	0.000311534	CD161+
TPT1	1.95E-07	0.269932619	0.957	0.939	0.002370387	CD161+
JUNB	3.41E-07	0.657633896	0.571	0.383	0.004153759	CD161+
RGCC	1.15E-06	0.78471183	0.467	0.307	0.014033602	CD161+
YPEL5	1.99E-06	0.706432785	0.476	0.332	0.024270061	CD161+
TUBA4A	5.30E-06	0.626318849	0.6	0.473	0.064545869	CD161+
RPL5	5.35E-06	0.309585874	0.89	0.874	0.065176357	CD161+
BTG1	3.28E-05	0.445015591	0.738	0.733	0.398948013	CD161+
VIM	7.38E-05	0.445111574	0.705	0.675	0.898724099	CD161+
ANXA1	9.94E-05	0.596054914	0.576	0.444	1	CD161+
DDIT4	0.000119219	0.701055478	0.424	0.321	1	CD161+
SLC38A2	0.000300906	0.547260643	0.4	0.282	1	CD161+
DUSP1	0.000379199	0.790788277	0.314	0.202	1	CD161+
CXCR4	0.000389842	0.591771413	0.51	0.448	1	CD161+
TAGLN2	0.000632756	0.428588031	0.59	0.549	1	CD161+
JUND	0.002319405	0.409562441	0.524	0.462	1	CD161+
HSPA8	0.002335772	0.418000112	0.533	0.462	1	CD161+
EEF1B2	0.003016906	0.323762206	0.643	0.632	1	CD161+
ZFP36L1	0.004403199	0.589065399	0.29	0.209	1	CD161+
FTH1	0.004790291	0.421106481	0.971	0.982	1	CD161+
RPL3	0.005594591	0.355777438	0.586	0.563	1	CD161+
CALM1	0.0056216	0.318878869	0.676	0.679	1	CD161+
PABPC1	0.006619021	0.381929822	0.624	0.61	1	CD161+
PTGES3	0.006784793	0.545600705	0.257	0.181	1	CD161+
ZNF331	0.006972405	0.521705729	0.333	0.26	1	CD161+
MT-ND2	0.007591401	0.400300715	0.552	0.549	1	CD161+

Supplementary Table 6. The correlation of CD8+PD-1+CD161- or CD8+PD-1+CD161+ T Cell distributon with clinical outcome

RFS

T region						N region					
Month	CD8+PD-1+CD161- T cells high	CD8+PD-1+CD161- T cells low	Month	CD8+PD-1+CD161+ T cells high	CD8+PD-1+CD161+ T cells low	Month	CD8+PD-1+CD161- T cells high	CD8+PD-1+CD161- T cells low	Month	CD8+PD-1+CD161+ T cells high	CD8+PD-1+CD161+ T cells low
20.2	1		37	1		84.7	0		34.2	1	
2.8	1		20.2	1		9.7	1		85.2	0	
1.9	1		81.9	0		34.2	1		37	1	
76.6	0		10.8	1		85.6	0		12.7	1	
2.7	1		89.3	0		1.4	1		87.6	0	
81.9	0		1.9	1		5.8	1		75.7	0	
1.4	1		2.8	1		87.6	0		9.7	1	
1.7	1		1.2	1		85.2	0		36.2	1	
1.2	1		80.8	0		2	1		85.6	0	
14	1		1.4	1		76.6	0		84.7	0	
34.2	1		1.7	1		70.9	1		36.3	1	
10.8	1		95.7	0		1.7	1		10.8	1	
70.9	0		34.2	1		27.3	1		84.2	0	
80.8	0		65.1	0		10.8	1		88.5	0	
5.2		1	77.1		0	75.7		0	27.3		1
65.1		0	5.2		1	36.2		1	1.4		1
77		0	97.5		0	1.9		1	2		1
9.7		1	36.3		1	36.3		1	14		1
97.5		0	14		1	55.3		1	76.6		0
2.2		1	2.7		1	14		1	70.9		1
19.1		1	75.7		0	84.2		0	1.9		1
37		1	76.6		0	77		0	55.3		1
89.3		0	70.9		1	1.7		1	1.8		1
95.7		0	77		0	12.7		1	5.8		1
77.1		0	9.7		1	2.2		1	1.7		1
36.3		1	2.2		1	37		1	2.2		1
75.7		0	19.1		1	88.5		0	77		0
77.9		0	77.9		0	1.8		1	1.7		1

OS

T region						N region					
Month	CD8+PD-1+CD161- T cells high	CD8+PD-1+CD161- T cells low	Month	CD8+PD-1+CD161+ T cells high	CD8+PD-1+CD161+ T cells low	Month	CD8+PD-1+CD161- T cells high	CD8+PD-1+CD161- T cells low	Month	CD8+PD-1+CD161+ T cells high	CD8+PD-1+CD161+ T cells low
33.4	1		49.5	1		84.7	0		54.5	1	
60.8	1		33.4	1		12.7	1		85.2	0	
3	1		81.9	0		54.5	1		49.5	1	
76.6	0		39.5	1		85.6	0		88.8	0	
5.9	1		89.3	0		4.1	1		87.6	0	
81.9	0		3	1		11.9	1		75.7	0	
4.1	1		60.8	1		87.6	0		12.7	1	
14.7	1		2.3	1		85.2	0		40.2	1	
2.3	1		80.8	0		94.3	0		85.6	0	
52.3	1		4.1	1		76.6	0		84.7	0	
54.5	1		14.7	1		80	0		48.4	1	
39.5	1		95.7	0		14.7	1		39.5	1	
80	1		54.5	1		30.3	1		84.2	0	
80.8	0		65.1	0		39.5	1		88.5	0	
11.3		1	77.1		0	75.7		0	30.3		1
65.1		0	11.3		1	40.2		1	4.1		1
77		0	97.5		0	2.9		1	94.3		0
12.7		1	48.4		1	48.4		1	52.3		1
97.5		0	52.3		1	59.6		1	76.6		0
4.9		1	5.9		1	52.3		1	80		0
31.2		1	75.7		0	84.2		0	2.9		1
49.5		1	76.6		0	77		0	59.6		1
89.3		0	80		0	12.9		1	3.8		1
95.7		0	77		0	88.8		0	11.9		1
77.1		0	12.7		1	4.9		1	14.7		1
48.4		1	4.9		1	49.5		1	4.9		1
75.7		0	31.2		1	88.5		0	77		0
77.9		0	77.9		0	3.8		1	12.9		1

Supplementary Table 7. 35 Mean expression of each marker in blood

Clusters	CD45	CD49d	CD11a	CD5	CD195	CD4	CD8a	CD7	CD25	CD134	CD2	CD95
T01	0.68	0.35	0.46	0.56	0.12	0.60	0.08	0.61	0.05	0.02	0.68	0.07
T02	0.80	0.69	0.70	0.22	0.29	0.26	0.25	0.17	0.04	0.05	0.51	0.43
T03	0.74	0.65	0.74	0.51	0.40	0.03	0.88	0.45	0.00	0.02	0.84	0.26
T04	0.77	0.54	0.70	0.41	0.52	0.01	0.12	0.63	0.02	0.02	0.77	0.23
T05	0.75	0.65	0.74	0.63	0.43	0.63	0.21	0.31	0.01	0.02	0.89	0.29
T06	0.76	0.44	0.52	0.59	0.15	0.03	0.85	0.71	0.01	0.02	0.73	0.06
T07	0.84	0.58	0.65	0.64	0.29	0.69	0.15	0.58	0.19	0.10	0.85	0.46
T08	0.77	0.52	0.63	0.57	0.22	0.07	0.05	0.48	0.02	0.03	0.71	0.34
T09	0.75	0.59	0.73	0.31	0.43	0.02	0.82	0.63	0.01	0.02	0.76	0.26
T10	0.77	0.46	0.61	0.62	0.23	0.66	0.13	0.58	0.16	0.06	0.81	0.38
T11	0.76	0.34	0.58	0.60	0.33	0.63	0.13	0.50	0.52	0.09	0.78	0.59
T12	0.79	0.40	0.52	0.62	0.16	0.66	0.13	0.71	0.06	0.02	0.71	0.11
T13	0.78	0.48	0.67	0.42	0.65	0.03	0.76	0.63	0.01	0.03	0.83	0.30
T14	0.73	0.64	0.73	0.44	0.36	0.03	0.87	0.50	0.01	0.02	0.81	0.24
T15	0.73	0.59	0.71	0.15	0.31	0.01	0.11	0.61	0.01	0.02	0.71	0.27
T16	0.75	0.52	0.74	0.30	0.29	0.02	0.20	0.50	0.03	0.03	0.72	0.19
T17	0.75	0.51	0.66	0.62	0.41	0.65	0.15	0.32	0.13	0.06	0.86	0.42
T18	0.77	0.57	0.68	0.51	0.44	0.04	0.85	0.57	0.04	0.02	0.84	0.35

Clusters	TIM_3	CD279	CD183	CD194	CD197	CD28	CD152	CD69	CD272	CD161	CD45RO	CD44
T01	0.02	0.00	0.00	0.54	0.58	0.33	0.16	0.00	0.07	0.01	0.03	0.32
T02	0.05	0.02	0.01	0.89	0.55	0.24	0.41	0.00	0.16	0.08	0.36	0.71
T03	0.03	0.07	0.00	0.76	0.23	0.06	0.17	0.25	0.02	0.05	0.51	0.47
T04	0.04	0.03	0.00	0.75	0.28	0.29	0.21	0.00	0.10	0.38	0.35	0.48
T05	0.06	0.11	0.00	0.76	0.28	0.14	0.28	0.00	0.04	0.14	0.59	0.50
T06	0.02	0.00	0.00	0.65	0.71	0.38	0.15	0.21	0.14	0.01	0.03	0.46
T07	0.06	0.07	0.00	0.78	0.59	0.64	0.35	0.00	0.13	0.50	0.64	0.59
T08	0.06	0.03	0.00	0.74	0.62	0.59	0.25	0.00	0.06	0.01	0.10	0.50
T09	0.02	0.01	0.00	0.77	0.25	0.07	0.19	0.19	0.04	0.28	0.04	0.46
T10	0.04	0.04	0.00	0.73	0.53	0.53	0.28	0.00	0.10	0.07	0.55	0.50
T11	0.04	0.03	0.00	0.82	0.45	0.57	0.31	0.00	0.03	0.03	0.63	0.52
T12	0.03	0.01	0.00	0.69	0.71	0.51	0.28	0.00	0.18	0.01	0.08	0.52
T13	0.04	0.05	0.00	0.73	0.30	0.51	0.22	0.12	0.07	0.65	0.47	0.50
T14	0.03	0.02	0.00	0.77	0.24	0.05	0.19	0.25	0.03	0.04	0.09	0.47
T15	0.03	0.01	0.00	0.75	0.23	0.06	0.18	0.00	0.03	0.24	0.06	0.44
T16	0.05	0.01	0.00	0.81	0.30	0.07	0.25	0.01	0.08	0.44	0.15	0.49
T17	0.06	0.10	0.00	0.75	0.39	0.50	0.28	0.00	0.06	0.13	0.59	0.51
T18	0.03	0.08	0.00	0.75	0.34	0.40	0.17	0.22	0.05	0.03	0.43	0.49

Clusters	CD27	CD278	CD45RA	CD3	CD9	CD57	CD137	HLA_DR	CD223	CD127	CD16
T01	0.47	0.10	0.73	0.78	0.10	0.01	0.00	0.01	0.00	0.15	0.04
T02	0.13	0.04	0.65	0.56	0.67	0.24	0.05	0.77	0.02	0.12	0.16
T03	0.06	0.09	0.22	0.76	0.37	0.83	0.22	0.15	0.00	0.07	0.10
T04	0.32	0.10	0.42	0.88	0.36	0.12	0.02	0.09	0.00	0.20	0.15
T05	0.01	0.16	0.23	0.76	0.39	0.82	0.21	0.15	0.00	0.16	0.09
T06	0.60	0.13	0.99	0.82	0.35	0.04	0.00	0.02	0.00	0.24	0.05
T07	0.57	0.28	0.29	0.88	0.42	0.05	0.01	0.04	0.00	0.49	0.07
T08	0.70	0.28	0.97	0.85	0.39	0.12	0.01	0.22	0.00	0.23	0.03
T09	0.10	0.04	1.00	0.77	0.35	0.32	0.03	0.14	0.00	0.06	0.15
T10	0.50	0.20	0.21	0.81	0.33	0.04	0.01	0.04	0.00	0.38	0.06
T11	0.54	0.41	0.17	0.82	0.34	0.04	0.01	0.22	0.00	0.03	0.05
T12	0.61	0.23	0.86	0.90	0.43	0.06	0.01	0.02	0.00	0.31	0.06
T13	0.30	0.11	0.32	0.81	0.41	0.05	0.01	0.05	0.01	0.42	0.07
T14	0.05	0.07	0.74	0.76	0.39	0.81	0.18	0.14	0.00	0.08	0.11
T15	0.06	0.05	0.97	0.78	0.27	0.55	0.08	0.16	0.01	0.05	0.11
T16	0.04	0.11	0.67	0.87	0.44	0.79	0.19	0.12	0.01	0.24	0.62
T17	0.16	0.16	0.17	0.77	0.33	0.06	0.01	0.10	0.00	0.35	0.07
T18	0.38	0.11	0.32	0.76	0.36	0.11	0.01	0.13	0.00	0.22	0.06

Normalized intensity

Supplementary Table 8. The abundance of 18 Clusters in blood

Clusters	P01	P02	P03	P04	P05	P06	P07	P08	P09	P10
T01	7.92	1.83	2.25	5.25	3	1.92	3.58	3.08	1.92	1.92
T02	0.25	0.08	0.17	0	0.08	0.17	0.33	0.08	0.42	0.17
T03	1	6.67	1.42	1	7.75	5.08	13.58	1.5	8.58	2.58
T04	7.17	2.5	16.17	6.33	9.42	1.25	3.5	7.33	11.92	3.67
T05	0.58	2.5	7.75	0.5	2.42	10.17	2.42	5.25	5.92	3
T06	11.33	3.5	6.25	4.83	1.58	1.17	6.25	5.67	4.67	3.5
T07	4.42	3.5	2.17	8.17	3.58	3.17	4.17	2.75	4.33	6.25
T08	0.25	0.17	0.08	0.17	0.17	0.25	0.5	0.5	0.25	0.25
T09	3.67	10.75	7	3	3.25	14.17	3.67	2.83	2.83	7.5
T10	10.67	11.67	6.33	18.17	8.58	11.5	11.67	11	12.83	16.67
T11	0.75	1.5	1.42	2	1.42	1.83	2.67	0.33	2.17	2.92
T12	17.42	5.58	9.67	11.92	10.92	3.58	6.33	5.17	4.75	4.5
T13	2.83	1.17	3.17	2	3.92	0.83	1	5.42	1.58	1.75
T14	6	14.58	6.58	4.33	19.5	8.5	5.67	4.5	10.92	6.08
T15	0.5	0.58	0.42	1.5	2.75	0.25	0.17	1.17	1.67	0.33
T16	1.92	1.83	15.08	5.42	9.08	0.5	0.5	2.42	2.67	5.08
T17	8.92	10.5	6.25	13.42	3.92	22.42	12.92	18.5	10.17	16.17
T18	14.42	21.08	7.83	12	8.67	13.25	21.08	22.5	12.42	17.67

Cell percentage(%)
